# Protein Electrostatics Tune the Singlet–Triplet Energy Gap in Natural and Engineered Phototropin Light-Oxygen-Voltage (LOV) Domains

**DOI:** 10.1021/jacs.5c21945

**Published:** 2026-02-04

**Authors:** Stephen O. Ajagbe, Paulami Ghosh, Samer Gozem

**Affiliations:** † Department of Chemistry, 1373 Georgia State University, Atlanta, Georgia 30302, United States

## Abstract

Photoactive flavoproteins such as Light-Oxygen-Voltage (LOV) domains serve as scaffolds for tuning the photophysics of their bound flavin cofactor through sequence mutations. This variable tuning effect has led to the development of a series of engineered LOV-based proteins that optimize fluorescence, intersystem crossing (ISC), photoreduction, and/or adduct formation over a range of time scales. To better guide future engineering efforts, we recently employed hybrid quantum mechanical/molecular mechanical (QM/MM) models of LOV domains to study how intradomain electrostatics exert control over flavin’s photophysics. This work focuses on a series of three LOV1 and three LOV2 domains from *Arabidopsis thaliana* (AtLOV), *Avena sativa* (AsLOV), and *Chlamydomonas reinhardtii* (CrLOV), as well as two engineered protein variants used for singlet oxygen generation, miniSOG and SOPP3. The results, which include an analysis of conformational flexibility, energetics of low-lying singlet and triplet π,π* and *n*,π* states, and protein electrostatic projection maps, shed light on the variations in ISC efficiency in those systems. We found that LOV1 domains are more flexible than LOV2 domains and have consistently higher triplet *n*,π* $\left(T_{n_{N},\pi^{*}}\right)$ energies when compared to the first optically active singlet π,π* $\left(S_{1\pi ,\pi^{*}}\right)$ state. This finding corroborates reports in the literature that ISC is typically less efficient in LOV1 domains. We also find that unfavorable triplet state energetics may provide an alternative explanation for a competing adduct formation directly from the singlet $S_{1\pi ,\pi^{*}}$ state in CrLOV2. In contrast, SOPP3 was found to have a smaller energy gap between the $S_{1\pi ,\pi^{*}}$ and $T_{n_{N},\pi^{*}}$ compared to miniSOG and all other natural LOV domains, which explains its improved ability to sensitize triplet oxygen. Together, these results emphasize the importance of electrostatic tuning in controlling the efficiency of ISC in LOV domains. The results also indicate that a heavy-atom effect alone cannot explain efficient ISC, especially in Cys-devoid systems like SOPP3.

## Graphical abstract

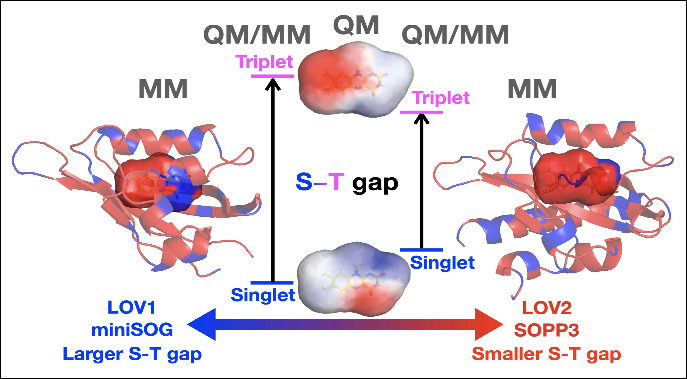

## Introduction

Flavins are versatile cofactors that participate in a wide range of biochemical processes.^1–7^ Although a subset of flavin-dependent proteins—hence called flavoproteins—are characterized by a covalent linkage between the protein and the flavin cofactor,^4,8^ most flavoproteins influence flavin electronic and chemical properties through noncovalent intermolecular interactions such as sterics and electrostatics. Steric effects arise from the spatial arrangement and size of amino acids surrounding the flavin, which influence their orientation and conformational flexibility as well as accessibility of substrates to the active site.^2,9^ Electrostatic interactions consist predominantly of charged or polar amino acid residues forming hydrogen bonds and/or salt bridges, or engaging in dipole–dipole interactions with flavin.^10^ In addition to shaping the protein structure and substrate interactions near flavin, electrostatics also directly influence the electronic properties of flavin and can effectively modulate its redox,^11–14^ photophysical,^15–18^ and spectral properties.^7,16,19,20^ Similar intermolecular tuning interactions have been demonstrated in various supramolecular systems, including proteins and solvents.^21–29^ For a planar and relatively rigid structure such as flavin in its oxidized form, electrostatic interactions are expected to play a more significant role than steric effects in tuning its electronic properties.

The role of protein electrostatics in tuning the photophysical properties of flavin is apparent in flavin-binding photoreceptor proteins such as LOV domains. In nature, LOV domains modulate a wide range of physiological responses to blue light (∼450 nm).^30–35^ In these proteins, flavin is responsible for blue-light absorption, upon which the chromophore becomes electronically excited and forms a covalent adduct with a highly conserved cysteine (Cys). Typically, this adduct formation proceeds via a triplet intermediate accessed by intersystem crossing (ISC) from the lowest singlet excited $\left(S_{1\pi ,\pi^{*}}\right)$ state.^36–47^ However, there have been a few reported instances, like in CrLOV2, where the adduct can form directly from the singlet as well as from the triplet state.^48^ This photochemical adduct formation induces structural and dynamic changes that lead to a cascade of varied responses, which have been leveraged for optogenetic applications.^30,31,49–51^

All plant phototropins characterized to date contain two flavin mononucleotide (FMN) binding LOV domains termed LOV1 and LOV2 based on their incidence in the protein sequence.^52,53^ While LOV2 is essential for signal activation, LOV1 modulates sensitivity, dimerization, and dynamic range, but is not required for light perception. Subsequent genomic and biochemical analyses have also revealed that LOV domains are present not only in plants, but are also distributed in bacteria, fungi, algae, and archaea, where they control a broad spectrum of physiological responses.^54–57^ Many of those are single-LOV proteins, unlike phototropins, with fungal LOV domains, such as VVD and WC-1, often employing flavin adenine dinucleotide (FAD) instead of FMN as the bound cofactor, and having distinct photochemical kinetics and redox properties.^54,55,58–61^

LOV domains also serve as important scaffolds for engineering different classes of fluorescent proteins (e.g., iLOV), singlet oxygen generators, and optogenetic tools.^62–68^ Engineering these functions into LOV domains relies largely on understanding protein modification of flavin photophysics. For instance, mutations that slow or prevent adduct formation suppress native function to introduce other potentially productive photophysical characteristics like fluorescence or long-lived triplet states. An example of LOV domain engineering for such desired characteristics is the development of genetically encodable singlet oxygen generators (SOGs). The first generation of such LOV-based SOGs includes miniSOG, an engineered variant with a relatively smaller quantum yield of 0.03 (at atmospheric conditions) compared to newer generations. A noteworthy member of the newer generations is SOPP3, which was engineered by mutations upon miniSOG and reported to have an oxygen sensitization quantum yield of 0.61.^69^

Despite the difficulty of accurately simulating properties such as ISC quantum yield and excited state lifetimes from first-principles, several spectroscopic and computational studies^10,36–48,70–91^ have provided insight into the mechanisms of ISC and covalent adduct formation in LOV domains. This is especially significant since ISC competes with other radiative and nonradiative decay processes.^46,70^ However, computational modeling can still provide important insight into the electronic structure of flavin and how it is affected by the protein environment, especially in processes like ISC that are slow enough that singlet and triplet state energetics are relevant. The prevailing hypothesis attributes enhanced ISC efficiency to the sulfur atom of the conserved proximal cysteine, which is believed to promote spin–orbit coupling via a heavy atom effect.^72,83,86,89,92–94^ However, this mechanism does not fully account for experimental observations. Notably, efficient ISC has been documented in SOGs devoid of the adduct-forming cysteine, such as miniSOG^95^ and SOPP3,^69^ indicating that the heavy atom contribution is not a prerequisite for transition to the triplet-state. Furthermore, the presence of a proximal cysteine does not universally correlate with efficient ISC among all flavoproteins. This suggests that the current understanding of ISC in LOV domains remains incomplete; factors beyond the heavy atom effect, such as local electrostatics, hydrogen-bonding interactions, protein rigidity, and conformational dynamics, likely play crucial roles in fine-tuning the photophysical properties of the flavin chromophore. A more comprehensive elucidation of these effects will be essential for improving our ability to tune LOV photochemistry and for the rational design of LOV-based photoreceptors and optogenetic tools with customized ISC behavior.

In this work, we focus exclusively on LOV1 and LOV2 domains of plant phototropins from three organisms, as well as two SOG derivatives, to allow for a systematic comparison of a series of related protein domains. Given the essential role of LOV2 in light perception, there is expected to be a higher evolutionary pressure to optimize ISC in LOV2 than in LOV1 domains. A systematic study elucidating the photophysical differences between LOV1 and LOV2 domains can help provide more evidence for this hypothesis. Specifically, using molecular dynamics (MD) and QM/MM calculations, we investigate the differences in the electrostatic environment and flavin excited-state energetics in LOV1 and LOV2 of *Arabidopsis thaliana* Phototropin 2 (AtLOV1 and AtLOV2), *Avena sativa* Phototropin 1 (AsLOV1 and AsLOV2) and *Chlamydomonas reinhardtii* Phototropin (CrLOV1 and CrLOV2), as well as miniSOG and SOPP3. In all models, we treat the lumiflavin (LF, Figure 1) moiety quantum mechanically, since it is responsible for flavin’s spectroscopic and photophysical properties. Other parts of the system, including the FMN ribose phosphate group, protein, solvent, and counterions, are treated at the MM level of theory.

**Figure 1:**
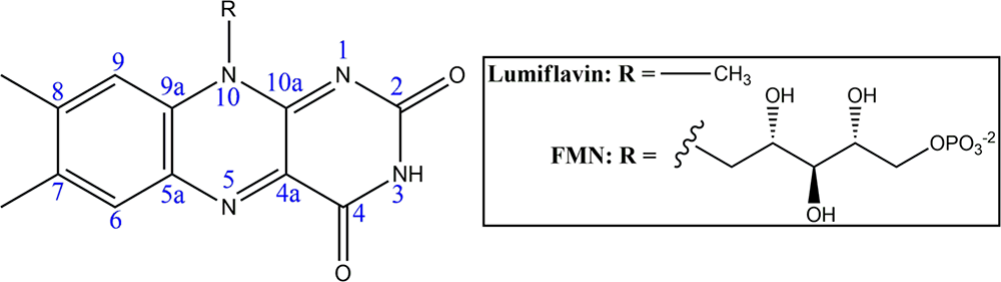
Isoalloxazine ring of FMN and atom number labels. R = CH_3_ for lumiflavin (LF) and R = ribose-5′-phosphate for FMN.

The first step of the LOV photocycle is blue or UVA light excitation. This ultimately populates the lowest singlet excited state, consistent with Kasha’s rule.^96^ It is then followed by ISC to the triplet state. Building on earlier computational work by Salzmann et al.,^88,89^ we recently found that this ISC step is likely driven by the $T_{n_{N},\pi^{*}}$ state which is energetically close to the first singlet excited state with $S_{1\pi ,\pi^{*}}$ character.^97^ While a triplet π,π* ( $T_{1\pi ,\pi^{*}}$ ) is ultimately populated and detected spectroscopically, potential energy scans indicated that it is unlikely that this state is involved in the initial ISC step.^97,98^

We also presented Electrostatic Spectral Tuning Maps (ESTMs) for both the $S_{1\pi ,\pi^{*}}$ and $T_{n_{N},\pi^{*}}$ states of the LF model. These 3D maps, shown in Figure 2, represent the energy shifts of those states relative to the ground (S_0_) state due to the presence of external charges. A comprehensive discussion of the ESTMs for the two optically active singlet excited states of LF ( $S_{1\pi ,\pi^{*}}$ and $S_{2\pi ,\pi^{*}}$ ) can be found in refs 20, 99 and that of $T_{n_{N},\pi^{*}}$ has been discussed in ref 97.

**Figure 2:**
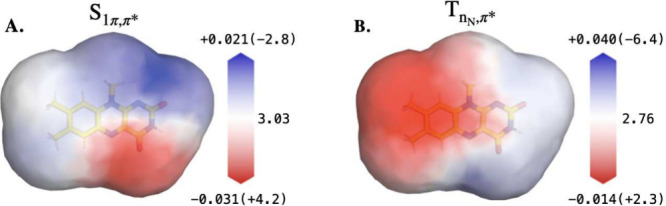
ESTMs for low-lying optically active $S_{1\pi ,\pi^{*}}$ and dark $T_{n_{N},\pi^{*}}$ states of LF, computed at the TD-B3LYP/cc-pVTZ level of theory. These represent the change in the vertical excitation energy of both states relative to the ground state (S_0_) energy due to the presence of a +0.1*e* probe charge placed at different positions on a surface that is two van der Waals (VdW) radii from each atom of LF. Red regions of the map indicate that positive charges in those regions would stabilize the specific excited state relative to the ground state (i.e., would red-shift the spectra for spectroscopic states). Blue regions indicate that positive charges in these positions would have the opposite effect.^99^ The effect of changing the VdW radius, the magnitude and sign of the probe charge, and computing the ESTM in the presence of other permanent point charges are discussed in ref 99. Additional testing of the effect of changing the VdW radius for both states in this figure is shown in SI Figure S5. The legends indicate the magnitude of the excitation energy shifts relative to the gas-phase reference excitation energy in eV (and in nm in parentheses): (A) $S_{1\pi ,\pi^{*}}$ , and (B) $T_{n_{N},\pi^{*}}$ . This figure is adapted with permission from panels A and D of ref 97, Copyright 2025 John Wiley and Sons.

In Figure 2A (ESTM of $S_{1\pi ,\pi^{*}}$ ), we show that the presence of positive charges around the lower halves of the pyrazine (central) and pyrimidine (right-most) rings of LF decreases the excitation energy of the $S_{1\pi ,\pi^{*}}$ state (i.e., red-shifts its absorption peak) whereas the presence of positive charges around the top halves of these same rings increases the excitation energy (i.e., blue-shifts its absorption peak).

Figure 2B shows that $T_{n_{N},\pi^{*}}$ is sensitive to electrostatic interactions either at the N_5_ lone pair or close to the hydrophobic side of LF. Most notably, the substantially different ESTMs of the $T_{n_{N},\pi^{*}}$ and $S_{1\pi ,\pi^{*}}$ states means that their relative energies can be strongly modulated by nearby charges or polar interactions. For instance, a positive charge or dipole near LF’s hydrophobic xylene (left-most) ring would red-shift the $T_{n_{N},\pi^{*}}$ while having almost no effect on the $S_{1\pi ,\pi^{*}}$ state. Meanwhile, a charge near the N_5_ atom or C_4_ carbonyl of flavin will have opposite effects on the stability of the $S_{1\pi ,\pi^{*}}$ and $T_{n_{N},\pi^{*}}$ states. These ESTMs, which are generated from quantum chemical calculations of LF in the gas phase, serve as a starting point for several of the discussions in this manuscript, and will be revisited in the context of the projection of LOV domain electrostatics on the flavin surface.

## Results

### Protein Dynamics and Active Site Analysis

Eight proteins are investigated in this work, five of which have crystal structures in the Protein Data Bank: AtLOV1 (PDB ID: 2Z6D), AtLOV2 (PDB ID: 4EEP), CrLOV1 (PDB ID: 1N9L), AsLOV2 (PDB ID: 2 V1A), miniSOG (PDB ID: 6GPU).^34,100–103^ The model of SOPP3 has a five point mutation difference from miniSOG, and was constructed by introducing those mutations into the miniSOG crystal structure using PyMOL, followed by minimization, equilibration, and MD steps. The two remaining models, AsLOV1 and CrLOV2, were constructed using AlphaFold 3 ^104^ based on sequences published in NCBI.^105,106^ The predicted local distance difference (pLDDT) scores per residue are shown in SI Figure S9.

These models were used as starting points for MD and QM/MM calculations using the Average Solvent Electrostatic Configuration (ASEC) approach (described in the Computational Details section), which has been applied for studying spectroscopic and photophysical properties of related flavoproteins, including AtLOV2, recently.^97^

Truncated models of the LOV1 and LOV2 domains (with 108 residues) were used for the MD simulations in this section only, to allow for direct comparison of the flexibility of analogous protein sequences having the same length. MiniSOG and SOPP3 have 115 residues and showed similar dynamic behaviors as the truncated LOV proteins. However, the full published PDB and/or NCBI models were used in the ASEC QM/MM calculations, as discussed below.

Across the board, LOV2 domain active sites appear more packed and less flexible than the corresponding LOV1 sites. For example, when using structures of the proteins generated after 5 steps of ASEC QM/MM optimizations, we find that LOV2 domains have more amino acids within 4 Å of the heavy atoms of isoalloxazine compared to the LOV1 domains (Figure 3). Specifically, from the ASEC/QM/MM optimized structures, we find that 16–17 residues are close (<4Å) to the flavin isoalloxazine ring in LOV1 domains, compared to 18–20 in LOV2 domains. MiniSOG and SOPP3 more closely resemble LOV2 in this case, since they also have 18–19 residues within 4 Å of the flavin isoalloxazine. In all cases, a key phenylalanine (Phe) residue in LOV2 is replaced by a nonaromatic residue in LOV1. This Phe group in LOV2 is normally right next to the flavin and extends from the hydrophilic side of the flavin to the central ring. We recently showed that this residue is partly responsible for a negative potential projected on the back side of the isoalloxazine ring onto the N_5_ atom (when viewed in the orientation shown in Figure 1) and may help stabilize the $T_{n_{N},\pi *}$ state that drives ISC in AtLOV2.^97^

**Figure 3:**
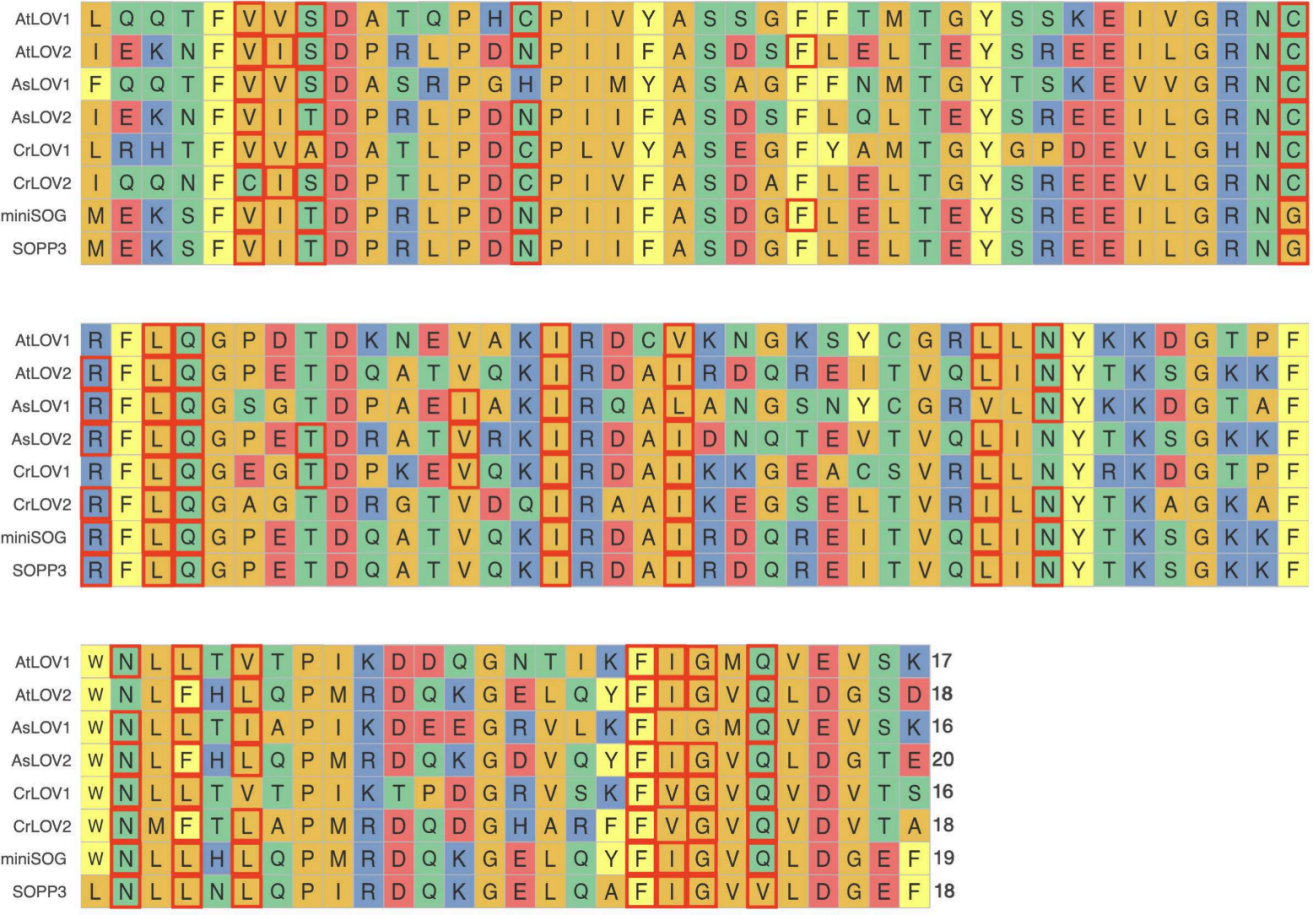
Sequence alignment carried out with ggmsa^107^ for 108 residues of truncated LOV1 and LOV2 proteins along with the corresponding segments from miniSOG and SOPP3. Residues within 4Å from the heavy atoms of the isoalloxazine ring, taken from QM/MM optimized structures of the six LOV domains and two SOG proteins, are highlighted with red boxes. The number of those proximal residues are indicated at the end of each sequence (e.g., 17 for AtLOV1). Distances of a few key residues close to the flavin N5 region are shown in Figure S6 in the SI, while all residues within 4Å are shown in Figure S7. The sequence alignments for the full (nontruncated) models are shown in Figure S10.

In Figure 4, we compare the RMSFs of the protein amino acid backbones. The structures are color coded blue (0.32 Å) to red (≥1.32 Å) to represent the RMSF of each amino acid relative to an average structure. We find that all LOV domains have the greatest mobility around the βE-βD loop, as well as at both the N and C termini, consistent with previous findings comparing CrLOV1 and AsLOV2.^110^ We also notice that, in general, LOV1 domains have increased flexibility in other regions, such as the βA-βB loops that are usually more rigid in LOV2 domains. For AtLOV1 and CrLOV1, we observe an above-average RMSF in the αB-α′A region. This increased rigidity in LOV2 is also clear when focusing on amino acids in the active site (within 4 Å of the flavin isoalloxazine ring, see Figure 5). Nomenclature of secondary structures is based on Figure 4g.^108^

**Figure 4:**
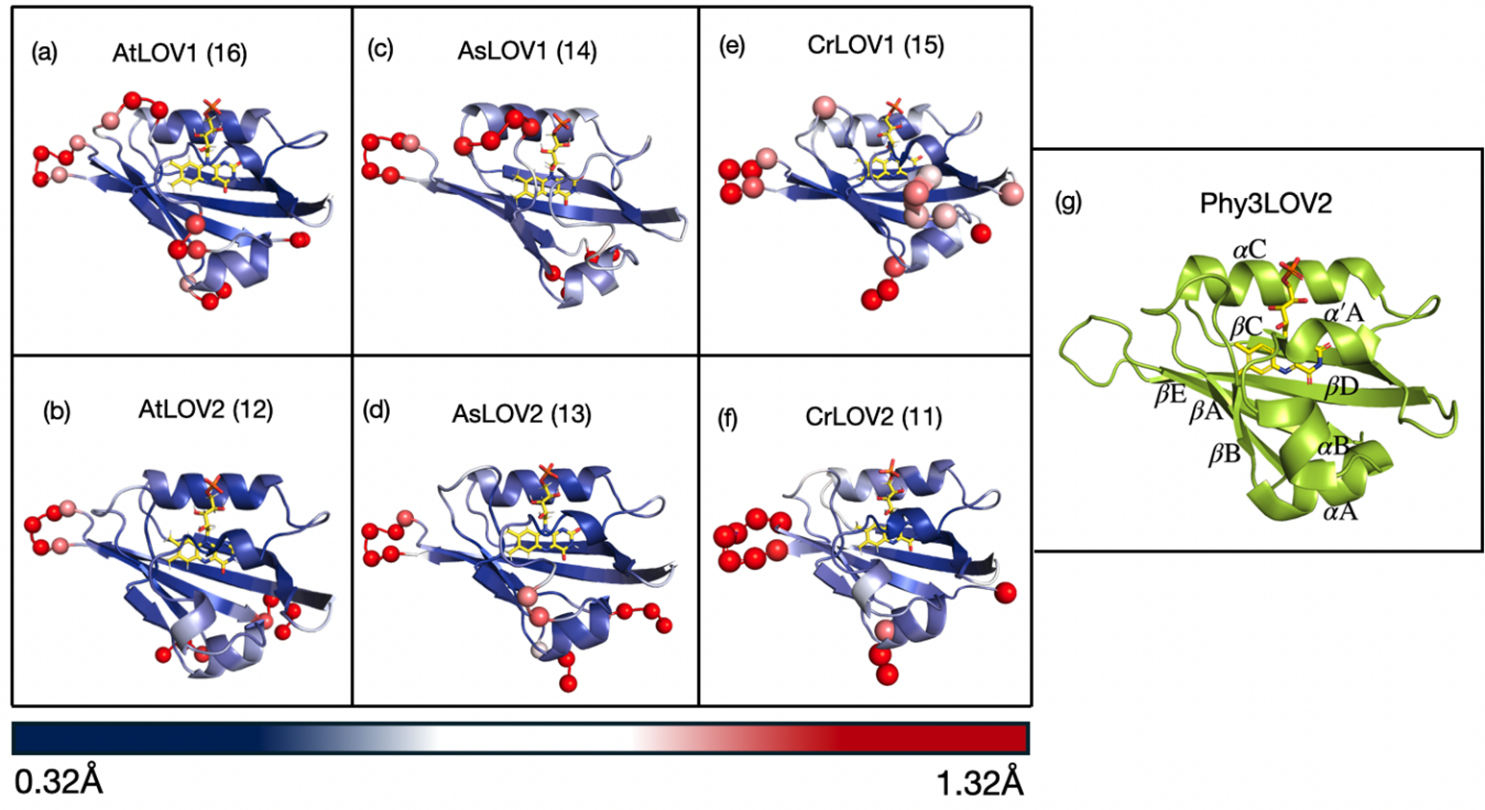
Structures of six LOV domains colored by the root-mean-square fluctuation (RMSF) per residue. The RMSF is calculated from 12,000 structures obtained over the span of 60 ns of MD simulations (two seeds of 30 ns each). The scale uses a RMSF range from 0.32 Å (dark blue) to 1.32 Å (dark red). Red spheres indicate the α-C of amino acids with RMSF above 0.82 Å. The numbers shown in the parentheses after each protein’s title are the number of spheres present for each system. Image (g) shows the structure of a prototypal LOV domain, *Adiantum capillus-veneris* Phytochrome 3 LOV2 domain (PDB ID: 1JNU), with secondary structure nomenclature consistent with refs 43, 108 . All images were generated using PyMOL.^109^

**Figure 5:**
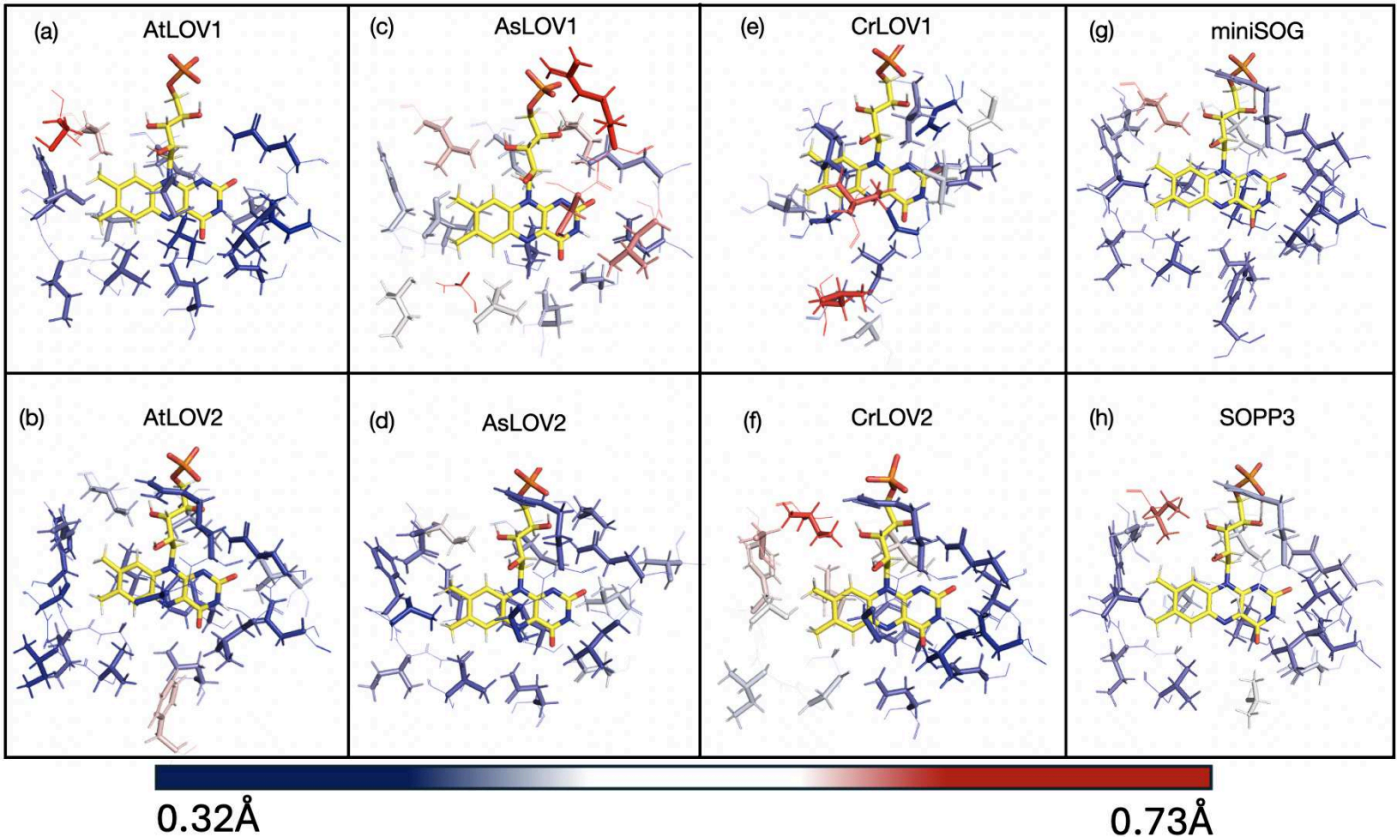
Structures of six LOV domains and two SOG proteins colored by the RMSF per residue within 4 Å. The RMSF is calculated from 12,000 structures obtained over the span of 60 ns of MD simulations (two seeds of 30 ns each). The scale uses a RMSF range from 0.32 Å (dark blue) to 0.73 Å (dark red).

Figure 5 generally shows increased rigidity (lower RMSF) for most residues around the flavin cofactor in LOV2 compared to LOV1. Most of this rigidity is near the hydrophilic pteridine part of the flavin, while the more flexible (greater RMSF) residues appear near the hydrophobic xylene part or phosphate group of the FMN.

### Electrostatic Projection Maps

Using the structures of each system obtained at the end of five steps of QM/MM ASEC optimization, we calculated the electrostatic potentials projected by the proteins onto the van der Waals (VdW) surface of LF (Figure 6). These images represent the electrostatic potential “felt” by the LF at a distance of 1 VdW radius from each heavy atom.

**Figure 6:**
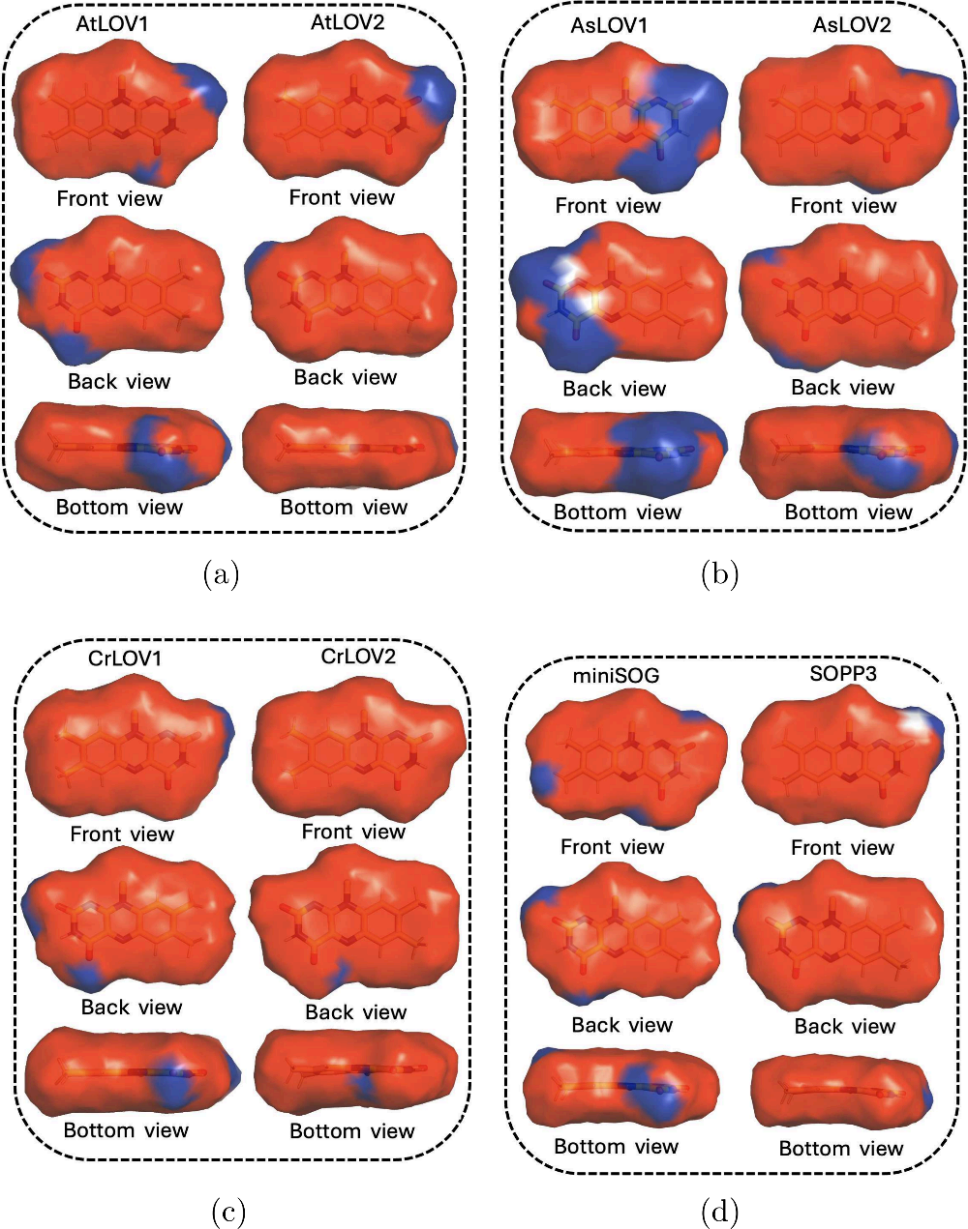
Projection of protein electrostatics onto the van der Waals surface of LF in the three LOV1 and LOV2 proteins: (a) AtLOV1 vs AtLOV2, (b) AsLOV1 vs AsLOV2, (c) CrLOV1 vs CrLOV2, and in (d) miniSOG vs SOPP3. Projections are colored based on the scale −0.25­(red) *k*T/*e* to 0.25­(blue) *k*T/*e*. In the top panels, the flavin is oriented as in Figure 1. In the center panels, the flavin is flipped such that the hydrophilic ring is on the left and the hydrophobic ring is on the right. In the bottom panel, we show the bottom view that highlights the electrostatic projection on the N_5_/C_4_ region.

We note that in a previous publication, we reported an electrostatic projection map for AtLOV2 on a surface that is 2 VdW radii from each heavy atom of the flavin. Here, we opt to use a 1 VdW radius surface because we found that it captures both long and short-range electrostatic interactions, while the 2 VdW radius surface overemphasizes short-range electrostatic interactions.

The total charge on LOV2 domains, after including the −2 charge on the FMN phosphate tail, is always negative (−2 for AtLOV2, −4 for AsLOV2, and −4 for CrLOV2). Meanwhile, the charge is usually positive for LOV1 domains (+3 for AtLOV1, +3 for AsLOV1, and 0 for CrLOV1). The charge on miniSOG is −4, while on SOPP3 it is −5. However, despite those differences in the total protein charge, we find a largely negative electrostatic potential (red region) from all proteins projected onto the LF, especially over the xylene and central rings of isoalloxazine. We also observe the conservation of a positive potential (blue region) around the C_2_/N_3_ and C_4_/N_5_ atoms across the board, except for LF in CrLOV2, where the positive potential around C_2_/N_3_ does not exist. On the other hand, AsLOV1 showed the highest positive potential (blue) in the hydrophilic region.

### Vertical Excitation Energies (VEEs) of the $S_{1\pi ,\pi^{*}}$ State

Table 1 presents excitation energies (in nm) computed using multireference QM/MM methods for the first optically active singlet excited state ( $S_{1\pi ,\pi^{*}}$ ) of LOV1 and LOV2 domains in the three organisms, and in the two SOGs studied. These calculations are compared to experimental UV/vis absorption maxima (in nm). In general, theoretical $S_{1\pi ,\pi^{*}}$ values range from 430 to 441 nm, and are systematically blue-shifted compared to the experimental values (439 to 448 nm). We have previously shown that theoretical vertical approximations overestimate the transition energies for the $S_{1\pi ,\pi^{*}}$ state of flavin when Franck–Condon factors (FCF) are not accounted for.^20,115–117^ However, the calculation of FCFs in a protein environment is not straightforward, so here we aim at systematically comparing VEEs with the assumption that FCFs are comparable in magnitude in all eight systems.

**Table 1:** VEEs, Reported in Units of Wavelength (nm), for the First Optically Active Singlet $S_{1\pi ,\pi^{*}}$ Excited State of LF in Various LOV Domains Computed Using 8-Roots (10,10) MS-CASPT2/ANO-L-VDZP Level of Theory^a^

	AtLOV1	AtLOV2	AsLOV1	AsLOV2	CrLOV1	CrLOV2	miniSOG	SOPP3
Theory	435 ± 1^i^	432 ± 2	441 ± 2	430 ± 1	432 ± 1	430 ± 1	440 ± 1	430 ± 1
Experiment	448^b^	447^c^	448^d^	447^d^	445^e^	445^f^	448^g^	439^h^

a The calculations are compared to the wavelength of maximum absorbance (*λ*_max_ obtained from experimental UV/vis spectra reported in the literature. The computed wavelengths and standard deviations are obtained from 4 steps of ASEC QM/MM calculations

b The experimental λ_max_ of $S_{1\pi ,\pi^{*}}$ for AtLOV1 was obtained by digitizing spectra from ref 111.

c The experimental λ_max_ of $S_{1\pi ,\pi^{*}}$ for AtLOV2 was obtained by digitizing spectra from ref 112.

d The experimental λ_max_ of $S_{1\pi ,\pi^{*}}$ for AsLOV1 and AsLOV2 were obtained by digitizing spectra from ref 42.

e The experimental λ_max_ of $S_{1\pi ,\pi^{*}}$ for CrLOV1 was obtained by digitizing spectra from ref 113.

f The experimental λ_max_ of $S_{1\pi ,\pi^{*}}$ for CrLOV2 was obtained by digitizing spectra from refs 48, 111 .

g The experimental λ_max_ of $S_{1\pi ,\pi^{*}}$ for miniSOG was obtained from ref 114.

h The experimental λ_max_ of $S_{1\pi ,\pi^{*}}$ for SOPP3 was obtained from ref 69.

i For AtLOV1 we obtained a standard deviation <0.5, which was rounded up to 1 nm.

In comparing the absorption peaks for each class, we find that the $S_{1\pi ,\pi^{*}}$ absorption peak is red-shifted in LOV1 compared to LOV2 across all 3 organisms. This effect is small but consistent in the experimental data (just 1 nm), while it is exaggerated in the QM/MM models, which, however, give the same trend.

In contrast to the LOV1 and LOV2 domains, miniSOG and SOPP3 show a larger variation in their absorption. MiniSOG has a λ_max_ of 448 nm, comparable to AtLOV1 and AsLOV1. On the other hand, SOPP3 has the largest blue-shift among the eight proteins, with a λ_max_ of 439 nm. The QM/MM calculations accurately predict this difference between miniSOG and SOPP3.

The NCBI entry for CrLOV2 includes the Jα helix, which is missing in most other models. To understand the effect of the Jα helix on the photophysics of flavin, we repeated the ASEC QM/MM calculations for CrLOV2 with and without the presence of this helix. The results, shown in Table S1 and Figure S11 of the SI, indicate that the CASPT2 computed excitation energies for the relevant singlet and triplet states are consistent in the two models, so the inclusion or omission of the Jα in the QM/MM model has a negligible influence on the results.

### VEEs of Low-Lying Singlet and Triplet Excited States of Flavin

Figure 7 represents the comparison of VEEs of LF in gas phase, aqueous solution (modeled using a polarizable continuum model, PCM), three LOV1 domains, three LOV2 domains, and two SOGs. Here, we increased the level of theory to incorporate excited states that include nonbonded *n*_N_ orbitals. The $S_{1\pi ,\pi^{*}}$ (blue) and $T_{1\pi ,\pi^{*}}$ (yellow) states exhibit π → π* character, which remains relatively stable across LOV domains, with energy values ranging from 2.68 to 2.75 eV and 2.11 to 2.19 eV, respectively. We find the same trend in VEEs of the $S_{1\pi ,\pi^{*}}$ state as observed in Table 1, with LOV1 domains and miniSOG generally slightly red-shifted relative to the LOV2 domains and SOPP3, despite the change in methodology here to incorporate *n*_N_ orbitals. The results for gas-phase, LF-PCM, and AtLOV2 here are comparable to the calculations carried out using 15-root state averaging in ref 97. While the LF-PCM model is understood to be a different level of approximation for the effect of solvation, it serves as a useful reference to the other QM/MM calculations.

**Figure 7:**
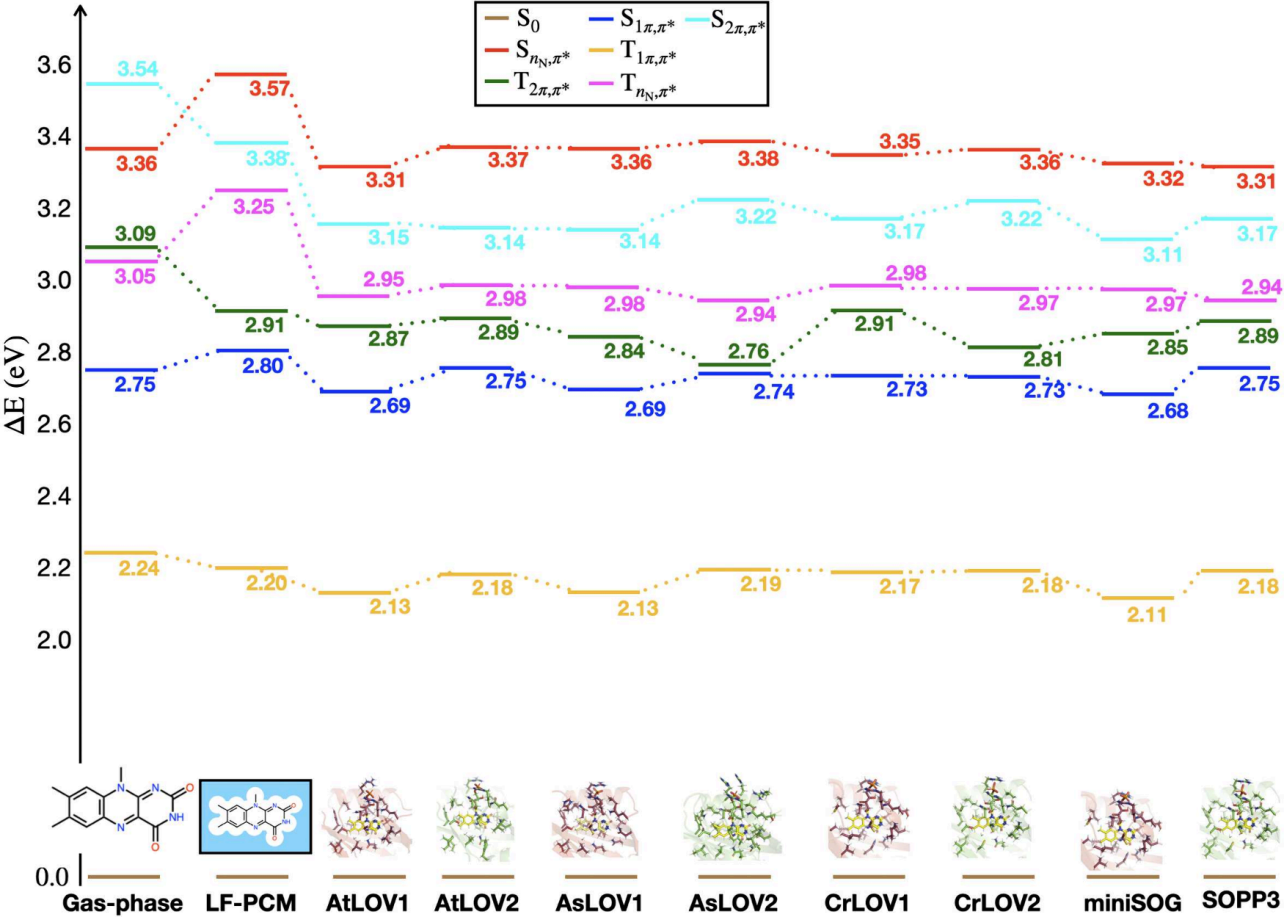
Comparison of VEEs (in eV) of LF in gas-phase, PCM-water, and in various LOV domains (AtLOV1, AtLOV2, AsLOV1, AsLOV2, CrLOV1 and CrLOV2, miniSOG and SOPP3) calculated at 15-roots (14,12) MS-CASPT2/ANO-L-VDZP level of theory: S_0_ (brown), T_1_ (yellow), $S_{1\pi ,\pi^{*}}$ (blue), $T_{n_{N},\pi^{*}}$ (magenta), $T_{\pi ,\pi^{*}}$ (green), $S_{n_{N},\pi^{*}}$ (red), $S_{2\pi ,\pi^{*}}$ (cyan).

The calculations show that embedding FMN within LOV domains leads to a stabilization of most excited states compared to gas-phase and implicit solvent models. However, there are slight differences between the eight proteins studied, suggesting minor variations in the electrostatic environment in each protein. Higher-lying singlet and triplet excited states such as $T_{2\pi ,\pi^{*}}$ (green), $T_{n_{N},\pi^{*}}$ (magenta), $S_{2\pi ,\pi^{*}}$ (cyan), and $S_{n_{N},\pi^{*}}$ (red) exhibit more significant fluctuations indicating differential stabilization depending on the LOV protein. The VEEs for higher-lying singlet and triplet π,π* excited states ( $S_{2\pi ,\pi^{*}}$ in cyan and $T_{2\pi ,\pi^{*}}$ in green) get red-shifted with respect to gas-phase LF and PCM-water model in agreement with findings by Salzmann et al.^88^

To isolate the effect of the LOV protein environment on the singlet–triplet energy, Figure 8 displays the difference in energy between the $T_{n_{N},\pi^{*}}$ and $S_{1\pi ,\pi^{*}}$ states. Figure 8 reinforces the conclusion that $\Delta_{verticalS_{1}-T_{n_{N},\pi^{*}}}E$ is smaller in LOV domains than for flavin in solution and in isolation. Furthermore, in all three organisms, the LOV2 domains have a smaller $\Delta_{verticalS_{1}-T_{n_{N},\pi^{*}}}E$ compared to their corresponding LOV1 domains, which implies that the protein environment of LOV2 domains either destabilizes the $S_{1\pi ,\pi^{*}}$ state, stabilizes the $T_{n_{N},\pi^{*}}$ state, or does both.

**Figure 8:**
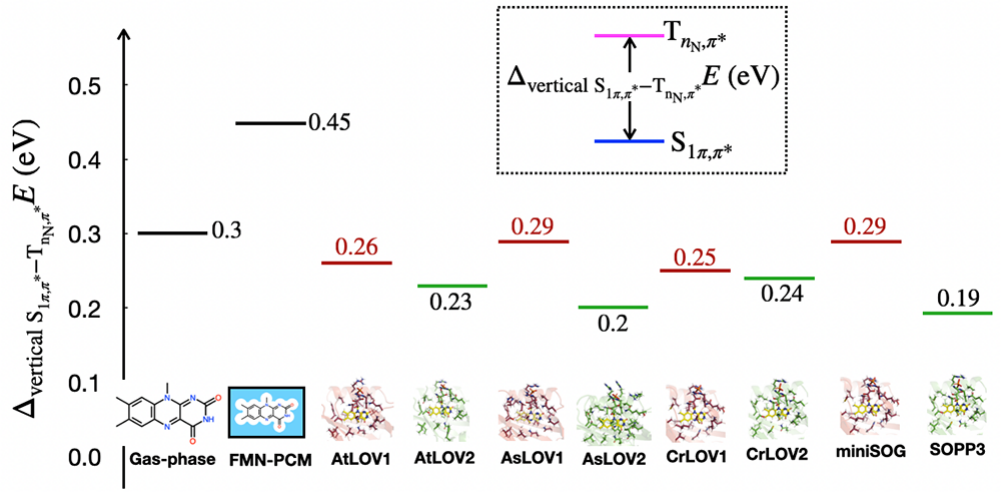
Comparison of $\Delta_{verticalS_{1}-T_{n_{N},\pi^{*}}}E$ (in eV) of LF in gas-phase, PCM-water and various LOV domains calculated at 15-roots (14,12) MS-CASPT2/ANO-L-VDZP level of theory.

Figure 8 also displays a large difference in $\Delta_{verticalS_{1}-T_{n_{N},\pi^{*}}}E$ when comparing miniSOG and SOPP3. While miniSOG has a relatively high singlet–triplet gap, comparable to AsLOV1, SOPP3 has the lowest $\Delta_{verticalS_{1}-T_{n_{N},\pi^{*}}}E$ among the proteins presented in this work.

### Pathway to the $T_{n_{N},\pi^{*}}$ State in LOV Domains

We constructed one-dimensional potential energy scans (1D-PESs) connecting the $S_{1\pi ,\pi^{*}}$ minimum to the minimum of the $T_{n_{N},\pi^{*}}$ state of flavin in all three classes of LOV1 and LOV2 domains. Figure 9 presents one of those scans, shown for AtLOV1. The scans for five other LOV1 and LOV2 domains are included in the SI Figure S8. The differences in the adiabatic excitation energies between the $S_{1\pi ,\pi^{*}}$ and $T_{n_{N},\pi^{*}}$ $\left(\Delta_{adiabaticS_{1}-T_{n_{N},\pi *}}\right)$ are obtained in a similar way to the PES from Figure 9. The energies are now reported at the TD-B3LYP/cc-pVTZ level of theory to mitigate issues with root switching or discontinuities in energy sometimes encountered with MS-CASPT2 along these scans.^97,118–120^

**Figure 9:**
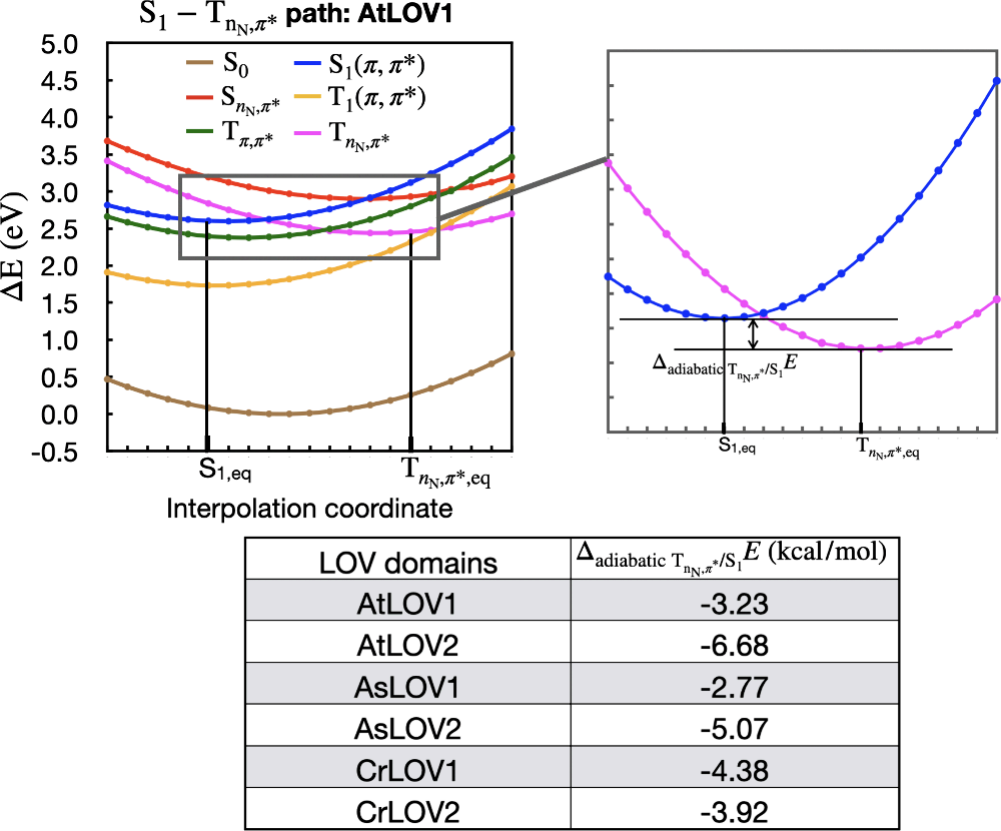
A representative TD-B3LYP/cc-pVTZ PES of LF in AtLOV1 connecting the $S_{1\pi ,\pi^{*}}$ minimum to $T_{n_{N},\pi^{*}}$ minimum. In this PES, ground state S_0_ (brown), and five low-lying excited states i.e. $S_{1\pi ,\pi^{*}}$ (blue), $S_{n_{N},\pi^{*}}$ (red), T_1_ (yellow), $T_{\pi ,\pi^{*}}$ (green) and $T_{n_{N},\pi^{*}}$ (magenta) have been shown to locate the potential crossings between low-lying excited states. The $\Delta_{adiabaticT_{n_{N},\pi *}/S_{1}}E$ for each LOV domains is tabulated below the PES.

## Discussion

The results in Figures 3, 4, and 5 all consistently show that LOV2 domains have a more packed (i.e., more amino acids within 4 Å of flavin’s isoalloxazine ring) and more rigid (lower RMSF) amino acid environment compared to LOV1. This packed and rigid environment is expected to be important for regulating flavin’s photophysics and funneling it toward the desired photochemistry, which implies that the increased flexibility of the protein environment near flavin contributes to a lower photophysical efficiency in LOV1 by allowing competing processes such as internal conversion to occur.^121^ MiniSOG and SOPP3 appear to be intermediate cases; they have more packed environment near the flavin, and a generally lower RMSF for those amino acids, but have more flexibility further away from the flavin binding site. A rigid environment with closely controlled electrostatics, such as in LOV2 and SOGs, can either increase the quantum yield of fluorescence (by electrostatically destabilizing the $T_{n_{N},\pi^{*}}$ state) or increase the quantum yield of ISC (by satisfying both conditions of minimal internal conversion and an energetically accessible $T_{n_{N},\pi^{*}}$ state).

Next, we focus on the effects of electrostatics. ESTMs shown in Figure 2 indicate that charges near C_4_/N_5_ have the largest potential to tune the relative energies of the $S_{1\pi ,\pi^{*}}$ and $T_{n_{N},\pi^{*}}$ states. For example, a positive charge near C_4_/N_5_ would destabilize $T_{n_{N},\pi^{*}}$ while simultaneously stabilizing $S_{1\pi ,\pi^{*}}$ (see also the legends in those figures, which indicate a total combined destabilization/stabilization of up to 0.071 eV by a + 0.1*e* probe charge at 2 VdW radii distance from the flavin in that region. This total combined destabilization/stabilization of 0.071 eV is based on the summation of the maximum red-shift in the VEE of the $S_{1\pi ,\pi^{*}}$ state, −0.031 eV, and maximum blue-shift in the VEE of the $T_{n_{N},\pi^{*}}$ state, 0.040 eV). Based on these maps, the more positive LOV1 projection near the N_5_/C_4_ region should stabilize the $S_{1\pi ,\pi^{*}}$ state and destabilize the $T_{n_{N},\pi^{*}}$ in comparison to LOV2 domains. Therefore, it is expected that, in general, LOV1 will have a more red-shifted absorption spectrum due to $S_{1\pi ,\pi^{*}}$ stabilization as well as a larger singlet–triplet gap. The maps also predict that miniSOG should behave more similarly to LOV1 domains while SOPP3 should behave more like LOV2 domains. Indeed, these predictions are confirmed by the multireference calculations in Table 1 and Figures 7 and 8, as well as by the TD-DFT ASEC QM/MM calculations in Figure 9. However, having an overly negative potential surrounding the flavin may start to destabilize the $T_{n_{N},\pi^{*}}$ in LOV2 domains, so there must be a delicate balance of negative and positive charges or dipoles surrounding the flavin.

A discussion on the effect of the protein environment on ISC would be incomplete without mention of the effect of hydrogen-bonding. H-bonds stabilize the electrons in the N_5_ lone pair, thus increasing the energy needed to excite them to the π* orbital. In other words, H-bonding destabilizes the $T_{n_{N},\pi^{*}}$ state and reduces ISC efficiency. Indeed, an investigation of H-bonding interactions near the flavin N_5_ region (see Figure S6 in the SI) indicates that there are no amino acids that form direct H-bonding interactions with the flavin N_5_ atom. The most likely candidate, which is the nearby glutamine, remains H-bonded to the O_4_ atom (with an average distance between heavy atoms ranging from an average of 2.9 to 4.1 Å in different LOV domains and SOGs) instead of H-bonding directly to the N_5_. A similar result was observed during MD simulations in iLOV, a LOV-based fluorescent protein.^14^ In the case of SOPP3, this key glutamine, which is generally conserved in all natural LOV domains, is replaced with a hydrophobic residue (valine), removing the potential of H-bonding altogether and contributing to a more negative potential near flavin’s N_5_. This hydrophobic environment can help explain the high efficiency of singlet oxygen generation in SOPP3. The next most likely candidate for H-bonding—the cysteine—maintains a distance that is at least 3.6 Å from the flavin’s N_5_. Therefore, none of the nearby side chains form a stable H-bond interaction directly with the N_5_ ensuring $T_{n_{N},\pi^{*}}$ stability and increased accessibility.

In LOV domains, the first step of the photocycle is population of the optically active $S_{1\pi ,\pi^{*}}$ state of flavin. Since ISC is typically most enhanced when a radiationless transition involves a change in the type of molecular orbitals (El-Sayed’s rule^122^), we expect that the low-lying $T_{n_{N},\pi^{*}}$ state is the most likely candidate for efficient ISC with $S_{1\pi ,\pi^{*}}$ . Indeed, LOV1, LOV2, and both SOGs all stabilize the $T_{n_{N},\pi^{*}}$ state (magenta in Figure 7) compared to the gas phase and PCM, in agreement with our earlier calculations for the AtLOV2 domain.^97^ The results in Figures 7 and 8 support the hypothesis that LOV domains drive efficient ISC by specifically stabilizing the $T_{n_{N},\pi^{*}}$ state relative to the spectroscopic $S_{1\pi ,\pi^{*}}$ state. Figures 7, 8, and 9 also all indicate that LOV2 better stabilize the $T_{n_{N},\pi^{*}}$ state relative to $S_{1\pi ,\pi^{*}}$ than LOV1 does. This stabilization is reflected in a smaller $\Delta_{verticalS_{1}-T_{n_{N},\pi^{*}}}E$ in the multireference calculations in Figure 8 and a more negative $\Delta_{adiabaticT_{n_{N},\pi *}/S_{1}}E$ in the TD-DFT calculations in Figure 9. The comparatively lower difference in $\Delta_{verticalS_{1}-T_{n_{N},\pi^{*}}}E$ is likely responsible for better access to the ISC region between $S_{1\pi ,\pi^{*}}$ and $T_{n_{N},\pi^{*}}$ states in LOV2 domains compared to LOV1, which would help explain the experimentally observed reduced photosensitivity of LOV1 domains compared to LOV2 domains.^42,123,124^

In the case of the two SOGs, the $\Delta_{verticalS_{1}-T_{n_{N},\pi^{*}}}E$ for SOPP3 is significantly lower than that computed for miniSOG. This difference correlates well with the observation that the quantum yield of oxygen sensitization in SOPP3 (0.61) is significantly higher than that of miniSOG (0.03) near standard conditions (21% oxygen at 23 °C).^69^

As discussed earlier, LOV2 domains and SOPP3 specifically project a more negative potential onto the flavin cofactor, especially near the N_5_ region (see Figure 6). Generally, in both LOV1 and LOV2 domains, there is a conserved positive potential near the carbonyl groups at C_2_ and sometimes near C_4_. Together, the negative protein potential near N_5_ and positive potential near C_2_ reduces the $\Delta_{verticalS_{1}-T_{n_{N},\pi^{*}}}E$ gap (as predicted by the ESTMs in Figure 2). For instance, in AsLOV1, which uniquely exhibits the strongest positive potential in the hydrophilic regions stretching from the C_2_/N_3_ to the C_4_/N_5_.

With QM/MM calculations, we observed that this positive potential around the N_5_/C_4_ atoms causes a redshift of the $S_{1\pi ,\pi^{*}}$ state in LOV1 domains relative to LOV2. These results appear consistent with a small (ca. 1 nm) but systematic redshift in experimental spectra of LOV1 and LOV2 domains reported in the literature. In contrast, in CrLOV2, there is a more negative potential across the entire isoalloxazine moiety, which extends all the way to the C_2_ carbonyl region. Such a negative potential near C_2_ will increase $\Delta_{verticalS_{1}-T_{n_{N},\pi^{*}}}E$ . These effects cause the VEEs of the two states relevant to ISC ( $S_{1\pi ,\pi^{*}}$ and $T_{n_{N},\pi^{*}}$ ) to be almost equal in the CrLOV1/CrLOV2 pair (the $\Delta_{verticalS_{1}-T_{n_{N},\pi^{*}}}E$ computed with MS-CASPT2 are within 0.01 eV of each other, while the TD-B3LYP $\Delta_{adiabaticT_{n_{N},\pi *}/S_{1}}E$ only differ by almost 0.02 eV, or 0.46 kcal/mol, see Figure 9). This too-negative electrostatic potential in CrLOV2, resulting in a small singlet–triplet energy gap, may explain why the Cys-flavin adduct formation occurs partially from the $S_{1\pi ,\pi^{*}}$ singlet state in this system, instead of occurring primarily from the $T_{n_{N},\pi^{*}}$ triplet state as reported in other LOV2 domains. However, we also acknowledge an alternative explanation that the competition between the singlet and triplet adduct formation may simply be kinetic, rather than thermodynamic, due to the proximity of the cysteine residue in CrLOV2 as suggested by Zhu et al.^48^

In summary, as discussed earlier for AtLOV2,^97^ we expect that the primary photophysical pathway follows these steps: blue light excites flavin from the S_0_ to the $S_{1\pi ,\pi^{*}}$ state, which, following vibrational relaxation, intersystem crosses to the $T_{n_{N},\pi^{*}}$ state. Flavin then undergoes internal conversion to the spectroscopically detected $T_{1\pi ,\pi^{*}}$ state.^36,97^ The PESs in Figures 9 and S8 in the SI support a similar mechanism for all 6 LOV domains. However, it is the subtle differences in the relative energetics of the singlet $S_{1\pi ,\pi^{*}}$ state and triplet $T_{n_{N},\pi^{*}}$ state that can explain the differences in the relative efficiencies of LOV domains and SOGs.

## Conclusion

In this study, we investigated the dynamics and photophysics of LF in three LOV1 (AtLOV1, AsLOV1 and CrLOV1) and LOV2 (AtLOV2, AsLOV2 and CrLOV2) domains as well as in two SOGs (miniSOG and SOPP3). We showed that the photophysics of LF in these proteins are at least in part governed by the electrostatic tuning of two key excited states: $S_{1\pi ,\pi^{*}}$ , and $T_{n_{N},\pi^{*}}$ . We also reveal distinct environmental sensitivities using ESTMs: positive potentials near the lower halves of the pyrazine/pyrimidine rings would redshift the $S_{1\pi ,\pi^{*}}$ state and blue-shift the $T_{n_{N},\pi^{*}}$ state, enabling selective energy modulation.

Investigating the electrostatic environments in LOV1 and LOV2 domains reveals a few differences consistent across all three organisms investigated. LOV2 domains generally project a more diffused negative electrostatic potential onto the flavin cofactor, especially over the xylene and central rings of isoalloxazine. This typically has the effect of reducing the singlet–triplet energy gap $\left(\Delta_{verticalS_{1}-T_{n_{N},\pi^{*}}}E\right)$ between the $S_{1\pi ,\pi^{*}}$ and $T_{n_{N},\pi^{*}}$ states, except in CrLOV2 where the negative potential extends up to the region near the C_2_ carbonyl and therefore gives a vertical gap similar to CrLOV1 (the 1D-potential energy scans even indicate that the $T_{n_{N},\pi^{*}}$ is more accessible from the $S_{1\pi ,\pi^{*}}$ state in CrLOV1 than in CrLOV2).

Together, these results further reinforce the hypothesis that ISC is driven by the $S_{1\pi ,\pi^{*}}$ and $T_{n_{N},\pi^{*}}$ states in LOV domains. They also indicate that subtle but systematic differences between LOV1 and LOV2 electrostatics may explain the improved efficiency of LOV2 domains, especially in AtLOV and AsLOV. Differences in CrLOV2 adduct formation mechanism and singlet oxygen generation efficiency in SOPP3 and miniSOG may potentially also be explained by electrostatic tuning of the singlet–triplet energy gap in those systems. While other factors, such as the proximity of the cysteine to the flavin in CrLOV2 or oxygen access to the flavin in SOGs, as well as differences in protein flexibility in LOV1 and LOV2, certainly contribute to details of the photophysical and photochemical mechanisms of those systems, electrostatic effects still play an important role. This indicates that there is an opportunity to tune photophysics of LOV domains and their variants through carefully designing the electrostatic environment, which is at least an important factor if not the central factor in determining flavin’s photophysics. Future efforts on LOV domains aimed at suppressing ISC may focus on more polar or H-bonding interaction near the flavin N_5_ region, and/or placing negatively charged residues or negative dipoles near N_1_/C_2_ region. Enhancing ISC is more challenging as it likely requires a delicate balance of polar and nonpolar interactions near flavin’s N_5_ and N_1_/C_2_ regions, but a good place to start is to further increase the hydrophobicity of amino acids near the N_5_, as done in SOPP3 through the mutation of N_5_-proximal glutamine to valine (useful if one does not intend to retain the native function of LOV domains).

This study also illustrates how QM/MM models may be applied to investigate sequence-dependent photophysics in LOV domains, which can guide future engineering of target systems such as fluorescent or singlet oxygen-generating proteins. The ASEC QM/MM protocol was recently updated, automated, and packaged as APEC-F 2.0 for the automatic construction of QM/MM models of flavoproteins for such purposes.^125^ Future work could also systematically benchmark the dependence of electrostatic contributions on QM region size by combining larger QM single-point TD-DFT calculations with enhanced MM sampling using modern force fields and water models. Such extensions would help quantify short-range polarization effects and conformational averaging while building directly on the current framework.

## Computational Details

### Electrostatic Spectral Tuning Maps (ESTMs)

Electrostatic Spectral Tuning Maps (ESTMs) were computed using the approach detailed in ref 99. A probe +0.1*e* point charge is moved on a surface that is at a distance of two VdW radii from each atom of LF. The coordinates on the van der Waals surface are determined using pyvdwsurface.^126^ At each probe position, TD-B3LYP/cc-pVTZ is used to calculate the excitation energy in the presence of that probe charge for each of the excited states shown in Figure 2. The points are represented using a color representing the change in excitation energy they induce in that state, and a color-coded ESTM is generated from those points using PyMOL.^109^ TD-B3LYP/cc-pVTZ singlet point calculations for generating the ESTMs were performed using Gaussian 16.^127^

### Electrostatic Projection Maps

Equilibrated and optimized protein crystal structures were obtained from the QM/MM calculations (specifically, from the fifth ASEC iteration), and electrostatic projection maps were computed by converting the PDB format to PQR format using PDB 2PQR with PropKa 3.1.^128,129^ This involved adding hydrogen atoms to the residues, assuming a pH of 7, with atomic charge and VdW radii parameters from the Amber force field.^130^

Crystallographic water molecules were deleted where present, and the structure subjected to the Adaptive Poisson–Boltzmann Solver (APBS)^131,132^ electrostatic potential calculation generating a 64 × 64 × 64 ${Å}^{3}$ cubic grid encapsulating the protein. Grid points were spaced 0.5 Å apart, resulting in a total of 2,146,689 grid points. Using the charges, positions, and VdW radii of the protein atoms in this grid, the APBS program was used to solve the Poisson–Boltzmann electrostatic equation for the grid points, populating each with the electrostatic potential at that point in 3D space. The resultant electrostatic map was saved in a dx file for further processing.

The coordinates of the heavy atoms of LF were obtained, and points were placed across the VdW surface 1 VdW radius from the LF’s heavy atoms using pyvdwsurface.^126^ At each point position, a KDTree was constructed for efficient neighbor search, and the average charge of the nearest 8 points in the grid was calculated as the potential at that point. These are shown as a surface above the isoalloxazine structures and colored in PyMOL with range −0.25 to 0.25 (see Figure 6).^109^

### 3D Structures

Crystal structures of LOV1 and LOV2 domains from *Avena sativa* Phototropin 1, *Arabidopsis thaliana* Phototropin 2, and *Chlamydomonas reinhardtii* Phototropin were obtained from the Protein Data Bank (PDB), and where unavailable, structures were modeled with Google’s AlphaFold 3 based on sequences from published ref 104. For Alphafold-generated 3D structures, FAD was used as the ligand and manually modified to FMN after structure generation. For crystal structures with multiple conformations of the photoactive cysteine, the conformation with the sulfur facing the xylene ring was selected, and for those containing multiple conformations of other amino acids with equal weight, conformation A was automatically selected.

### *Arabidopsis thaliana* (At) Phototropin 2

AtLOV1 structure was obtained from the PDB database (PDB ID: 2Z6D, 2.00 Å),^100^ and the sequence compared with NCBI Genbank ID: AAC27293.2, while AtLOV2 structure, (PDB ID: 4EEP, 1.70 Å),^133^ was compared in sequence with NCBI Genbank ID: AAC27293.2, confirming the sequence previously aligned by Crosson, Rajagopal and Moffat.^74^

### *Chlamydomonas reinhardtii* (Cr) Phototropin

CrLOV1 structure was obtained from the PDB database (PDB ID: 1N9L, 2.00 Å),^101^ and the sequence was compared with NCBI GenBank ID: CAC94940.1. For CrLOV2, a reference sequence region from Supporting Information of ref 102 was crosschecked with NCBI GenBank ID: CAC94940.1, and a 3D structure of this sequence was modeled using AlphaFold 3.^104^

### *Avena sativa* (As) Phototropin 1

AsLOV1 3D structure was based on sequence alignment of the CrLOV1 sequence from the PDB with GenBank Accession AAC05083.1, with the single addition of a Lysine at the C-terminal. Therefore, residues 133–241 of the sequence of Accession AAC05083.1 were modeled using AlphaFold 3. The sequence obtained from GenBank had an identical sequence to the one reported by Crosson, Rajagopal, and Moffat in the ref 74 but with 2–3 additional amino acids at the C and N termini. The AsLOV2 structure was obtained from the PDB database, AsLOV2 (PDB ID: 2 V1A, 1.65 Å),^103^ and the sequence was compared with NCBI GenBank ID: AAC05083.1.

### 4 Å Residue Selection

Residues within 4 Å of the LF structures were selected by displaying the proteins in PyMOL, and the LF heavy atoms were manually selected.^109^ This was further expanded to include residues within 4 Å, then saved as PDB.

### QM/MM Protocol

The QM/MM approach employed in this study builds on the Average Solvent Electrostatic Configuration (ASEC) method, which was initially developed to compute molecular properties within a statistically averaged solvent environment.^134,135^ This method has since been adapted for retinal-binding proteins^136,137^ and flavin-binding proteins.^16,97,125,138,139^ ASEC is designed to integrate the high accuracy of multireference quantum chemical calculations with the efficient conformational sampling afforded by molecular dynamics simulations.

FMN parameters were initially sourced from the AMBER parameter database maintained by the University of Manchester.^140^ To prepare the system, excess crystallographic water molecules were removed using the Dowser program,^141^ and charge neutrality was achieved by adding counterions. The resulting structure was used for MD simulations and for generating the ASEC environment. In this setup, quantum chemical calculations are carried out within a time-averaged electrostatic potential that encompasses contributions from the protein, solvent, and counterions. Although the term “solvent” in the ASEC acronym is used, it refers to this entire MM environment—including the protein and ionic components in addition to the explicit solvent.

The time-averaged electrostatic potential of the protein was derived from MD simulations performed with GROMACS.^142^ For each system, the protein was placed in a 7.0 nm × 7.0 nm × 7.0 nm cubic solvent box with periodic boundary conditions. Long-range electrostatic interactions were treated using the smooth particle-mesh Ewald (sPME) method, applying a cutoff of 1.2 nm for both Coulomb and van der Waals interactions. The AMBER99SB force field^143,144^ was used for the protein, and the TIP3P model^145^ was used for water molecules. The MD simulation protocol included three stages: a 300 ps heating phase from 0 to 300 K at 1 atm, followed by 4700 ps of equilibration, and finally a 5000 ps production run in the NPT ensemble under standard conditions. To construct the ASEC environment, 100 configurations were extracted at 50 ps intervals from the production trajectory. These snapshots were then merged to create an ensemble representation, with atomic charges and Lennard-Jones parameters scaled accordingly. The resulting structure was converted into Tinker format for subsequent QM/MM calculations.

The computational methodology integrates OpenMolcas^146^ with Tinker^147^ via an additive QM/MM scheme using electrostatic embedding. This setup accounts for both Lennard-Jones and electrostatic interactions through the Electrostatic Potential Fitted (ESPF) method.^148^ System preparation and molecular dynamics simulations were carried out using a combination of tools, including PropKa 3.1 ^129^ for p*K*_a_ prediction and protonation state assignment, Dowser^141^ for removing excess crystallographic waters, SCWRL4.0 ^149^ for side-chain optimization, and GROMACS^142^ for MD simulations. The protein system is partitioned into two regions: the QM region, which includes the LF chromophore, and the MM region, consisting of the rest of the system—namely, the protein, solvent, solution ions, and the ribose-5′-phosphate group. A hydrogen link atom (LA)^150^ is used to cap the QM region (see Figure S1 in the Supporting Information). To avoid overpolarization of the QM wave function, the atomic charges of MM atoms near the link atom are set to zero and their charges are redistributed to neighboring atoms. The ASEC protocol follows an iterative process that alternates between QM optimization and MD simulation. In each iteration, the QM subsystem is optimized within a fixed ASEC MM environment using electrostatic embedding. Based on the updated geometry and ESPF charges of the QM region, a subsequent 5 ns MD simulation is performed to generate a new ASEC environment. This cycle is repeated until the excitation energies converge, defined as four successive iterations yielding excitation energies within 0.02 eV of one another.

QM/MM geometry optimizations of the QM subsystem were performed using the (10,10) CASSCF/ANO-L-VDZP level of theory. No state-averaging was used for the ground state optimization. The (10,10) active space consists of five sets of π and π* orbitals shown in the SI (Figure S2).

### Vertical Excitation Energies (VEEs)

The VEEs of the $S_{1\pi ,\pi^{*}}$ state of LF in three classes of LOV1 and LOV2 domains and in miniOSG and SOPP3 were computed from the QM/MM optimized QM subsystem for each step using 8-roots MS-CASPT2/ANO-L-VDZP, with an imaginary level shift of 0.2 and no IPEA shift. Specifically, the average excitation energy and standard deviation of the VEEs of the $S_{1\pi ,\pi^{*}}$ state are calculated from the last four consecutive steps of QM/MM optimization. As the $S_{1\pi ,\pi^{*}}$ state is optically active π,π* in nature, we have used the (10,10) active space consisting of the same sets of π and π* orbitals (shown in Figure S3) as used for S_0_ optimization.

VEEs of all three classes of QM/MM optimized LOV1, LOV2 proteins, miniSOG and SOPP3 were calculated using the geometry of the last step with 15-roots (14,12) MS-CASPT2 level of theory. The (14,12) active space consists of two added nonbonding orbitals having electron density on N_1_ and N_5_ atoms of LF (i.e., the *n*_N_ orbitals) and the same sets of five π and π* orbitals used in (10,10) active space. The details about choosing (14,12) active space and the number of roots used in state-averaging has been discussed in refs 97, 98 . Nonbonding orbitals on the oxygen atoms are excluded from the active space because they are not expected to participate in flavin’s UV/vis photophysics. In a previous benchmark study,^98^ we found that the energetics of LF’s low-lying excited states are relatively insensitive to the number of states used in the state averaging in MS-CASPT2’s zeroth-order wave function, as long as the number of states is larger than two. Whenever a (14,12) active space is used, 15-roots state-averaging was also used to stabilize states involving nonbonding orbitals. The (14,12) active space and the orbitals involved in the low-lying excitation of LF in all the LOV domains studied here are shown in the SI (Figure S4).

### Potential Energy Scans (PESs)

We optimized the $S_{1\pi ,\pi^{*}}$ and $T_{n_{N},\pi^{*}}$ states of LF in all LOV1 and LOV2 domains keeping the S_0_ MM charges fixed. $S_{1\pi ,\pi^{*}}$ and $T_{n_{N},\pi^{*}}$ states were optimized using 3-roots (10,10) SA-CASSCF/ANO-L-VDZP and 15-roots (14,12) SA-CASSCF/ANO-L-VDZP level of theories, respectively. To locate potential crossings between these low-lying excited states, we then constructed one-dimensional PESs (1D-PESs) by a linear interpolation of Cartesian coordinates. The 1D-PESs connect the $S_{1\pi ,\pi^{*}}$ to the minimum of the $T_{n_{N},\pi^{*}}$ state ( $S_{1\pi ,\pi^{*}}$ - $T_{n_{N},\pi^{*}}$ path) of LF in LOV1 and LOV2 proteins. We added additional points on either side of each path through a linear extrapolation, generating a total of twenty-one geometries obtained through interpolation and extrapolation. Though the wave function-based multireference methods are more appropriate for locating potential crossings between excited states, in our recent study,^97^ we noticed that multireference methods such as MS-CASPT2 show a kink or discontinuity at least in one point of the PESs, but TD-B3LYP does not. Furthermore, TD-B3LYP was carefully benchmarked against multireference methods both in a gas-phase model^98^ and in AtLOV2.^97^ It was found that TD-B3LYP typically overstabilizes the $T_{n_{N},\pi^{*}}$ state relative to MS-CASPT2, but generally gave the same trend as MS-CASPT2. Therefore, in this study, we used TD-B3LYP/cc-pVTZ to compute the 1D-PESs of LF in all three classes of LOV1 and LOV2 domains. A representative TD-B3LYP/cc-pVTZ 1D-PES of AtLOV1 along with a tabulated form of $\Delta_{adiabaticT_{n_{N}},\pi^{*}}E$ in kcal/mol for all three LOV1 and LOV2 have been shown in Figure 9 and the 1D-PESs of rest of the LOV1 and LOV2 domains are given in Figure S8 in the SI.

All the wave function-based multireference calculations (MS-CASPT2, SA-CASSCF, and CASSCF) were performed with the ANO-L-VDZP basis set and using OpenMolcas version 22.10.^146^ Single-point calculations for 1D-PESs of LF with TD-B3LYP/cc-pVTZ method were performed using Q-Chem 6.0.2.^151^

### MD Analysis

Selected 100 frames used in 5 consecutive ASEC iterations were combined to form a 500 frame trajectory descriptive of protein dynamics. Waters were removed and the structure converted to PDB format for further analysis. Using MDAnalysis,^152,153^ the RMSF of C_α_ per residue was calculated and entered as the B-factor for each residue. This factor was normalized such that the maximum is 1 and the minimum is 0, and saved in a separate PDB file. This was colored in PyMOL using the spectrum b function, with range 0 to 1. Residues with normalized RMSF above average were selected, and their C_α_ atoms were shown as spheres.

For the 4 Å analysis, the residues known to be within 4 Å from the section above and shown in Figure 3 were selected from the PDB file containing RMSF data prior to normalization. The range of these was recorded, and they were colored according to the max and min C_α_ RMSF for each LOV1-LOV2 pair.

## Supplementary Material



## Data Availability

Data for visualizing ESTMs, EPMs and RMSF Analysis is found at https://github.com/gozem-gsu/LOV1-v-LOV2.git

## Notes

QM/MM partition scheme. Active space orbitals used in SA-CASSCF and MS-CASPT2 calculations. Electrostatic spectral tuning maps with probe charge placed at different positions on the 1×, 2×, and 3× VdW radii surface of LF. Protein structures showing the average bond distances between N_5_ of FMN and CYS, GLN and LEU (in case of LOV1) and PHE (in case of LOV2). Protein structures showing nearby residues within 4 Å . TD-B3LYP/cc-pVTZ PESs of various LOV1 and LOV2 domains: path to $T_{n_{N},\pi^{*}}$ state. Alphafold structures and pLDDT scores for CrLOV2 and AsLOV1. Full protein sequences of 8 proteins in this study. Comparison of CrLOV2 models with and without J’α helix. (PDF)
