# Supplementary material for: Protein Electrostatics Tune the Singlet–Triplet Energy Gap in Natural and Engineered Phototropin Light-Oxygen-Voltage (LOV) Domains
Source: J Am Chem Soc. 2026 Feb 4;148(7):7707–22. doi: 10.1021/jacs.5c21945 (PMC12951445; doi:10.1021/jacs.5c21945)
Supplement: Supplementary file 1 [file ja5c21945_si_001.pdf]

Supporting Information for:
Protein electrostatics tune the singlet-triplet energy gap in natural and engineered Phototropin LOV domains

Stephen O. Ajagbe[#,†], Paulami Ghosh[#,†] Samer Gozem[⋆,†]

*Email: sgozem@gsu.edu
# These two authors contributed equally to this work.
[†]Department of Chemistry, Georgia State University, Atlanta, Georgia, 30302, USA.

S1 QM/MM partition scheme

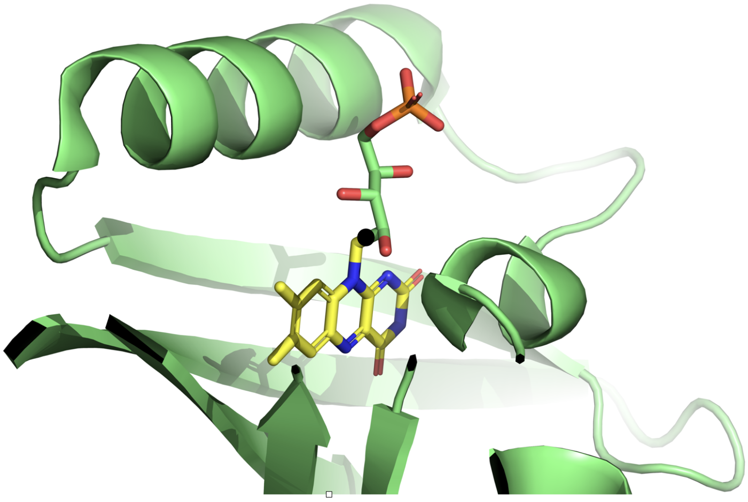

Figure S1: FMN inside the binding pocket of a LOV protein. This is a representative image to show the QM/MM partition. The black circle indicates the hydrogen link atom (LA), which separates the QM subsystem (LF, in yellow) and the MM subsystem (the ribose-5′-phosphate group and protein, in green). Reproduced with permission from Ghosh *et al.*, Chem. Eur. J. 31(22), e202500117, 2025. Copyright 2025, John Wiley and Sons.

S2 Active space orbitals used in SA-CASSCF and MS-CASPT2 calculations

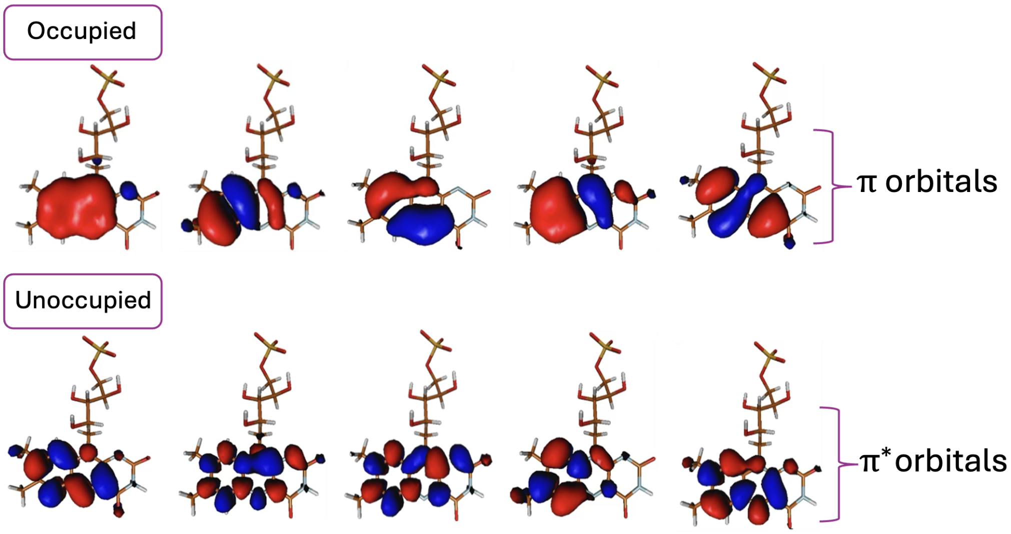

Figure S2: (10,10) active space for S_0 optimization for six LOV domains studied here.

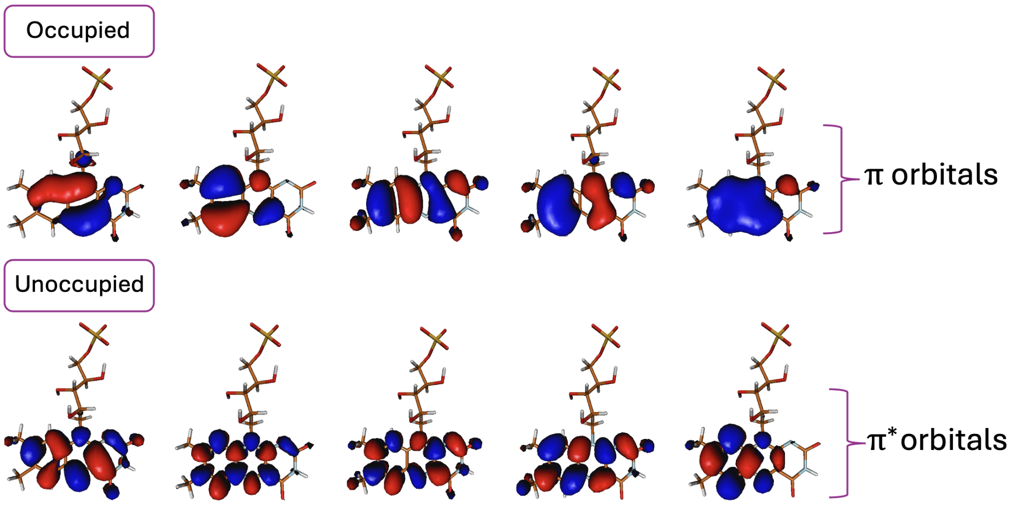

Figure S3: 8-roots (10,10) and 3-roots (10,10) active spaces used for VEEs of $S_{1\pi,\pi^*}$ spectra and optimization of $S_{1\pi,\pi^*}$ state of six LOV domains, respectively.

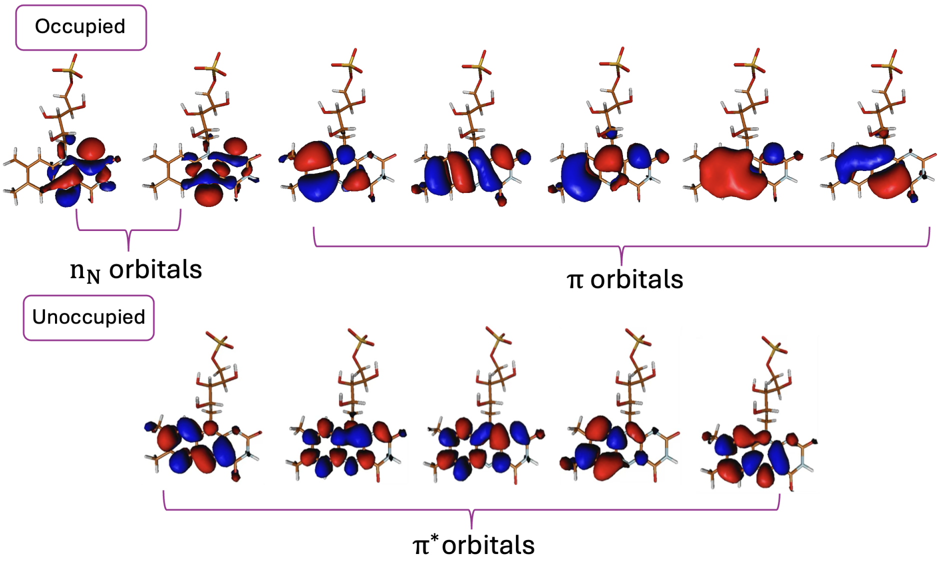

Figure S4: 15-roots (14,12) active space used for optimization of T_{n_N,π^*} and VEEs of low-lying singlet and triplet excited states for six LOV domains studied here.

S3 Electrostatic spectral tuning maps

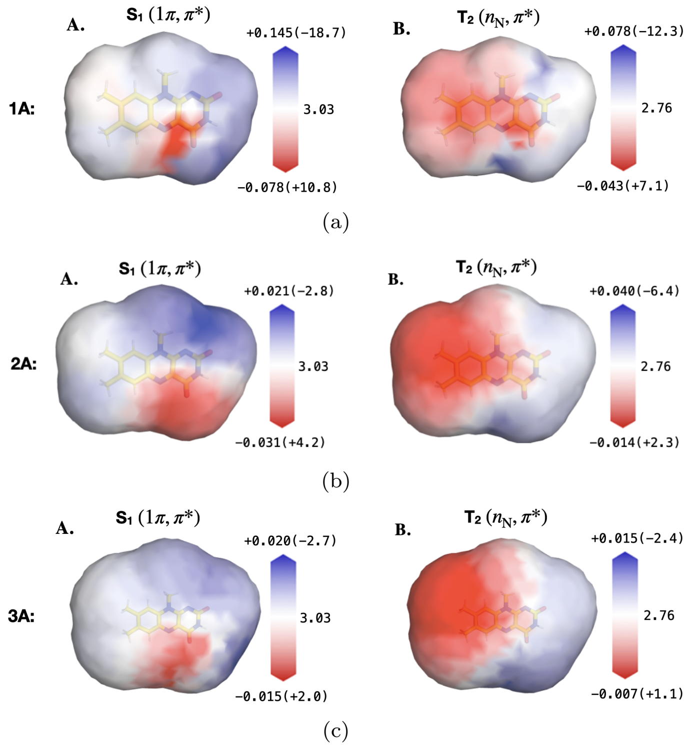

Figure S5: ESTMs for low-lying optically active $S_{1\pi,\pi^*}$ and dark T_{n_N,π^*} states of LF, computed at the TD-B3LYP/cc-pVTZ level of theory. The maps represent the change in the vertical excitation energy of these states relative to the ground state (S_0) energy due to the presence of $+0.1e$ probe charge placed at different positions on a surface that is (a) one, (b) two and (c) three Van der Waals (VdW) radii from each atom of LF.

S4 Six LOV domains showing the average bond distances between N_5 of FMN and CYS, GLN and LEU (in case of LOV1) and PHE (in case of LOV2

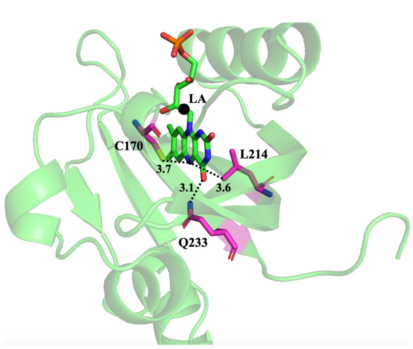

(a) AtLOV1

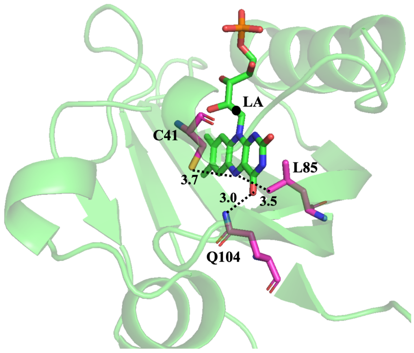

(b) AsLOV1

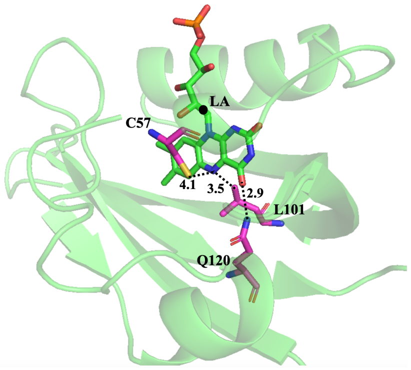

(c) CrLOV1

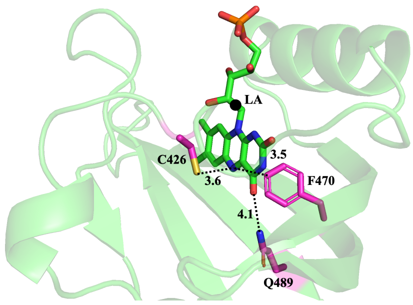

(d) AtLOV2

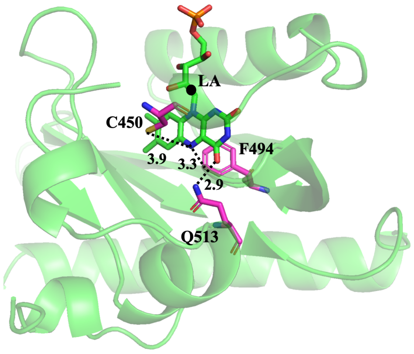

(e) AsLOV2

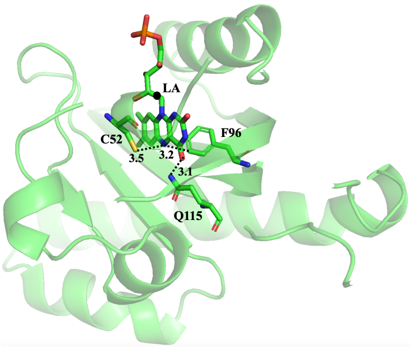

(f) CrLOV2

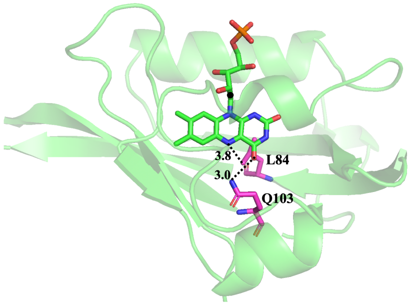

(g) miniSOG

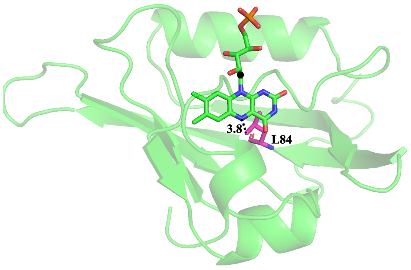

(h) SOPP3

Figure S6: Average bond distances between N_5 of FMN and the nearby Cys, N_5 of FMN and nearby Gln side chain nitrogen, and the distance between N5 of FMN and the carbon of a nearby hydrophobic residues for (a) AtLOV1 (b) AsLOV1 (c) CrLOV1 (d) AtLOV2 (e) AsLOV2 (f) CrLOV2, (g) miniSOG and (h) SOPP3.

S5 Six LOV domains showing nearby residues within 4Å

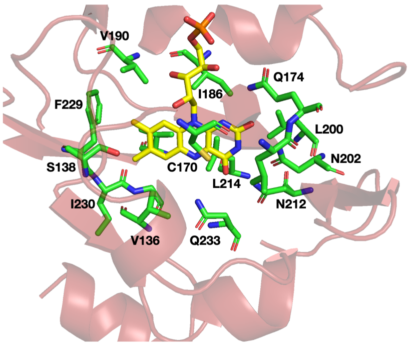

(a) AtLOV1

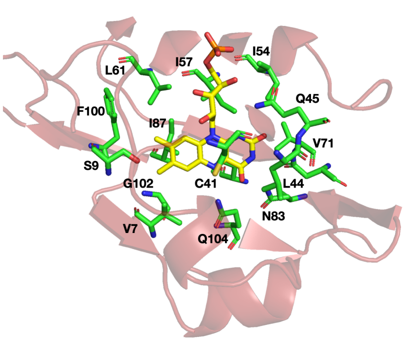

(b) AsLOV1

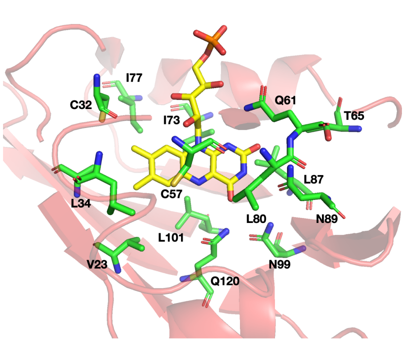

(c) CrLOV1

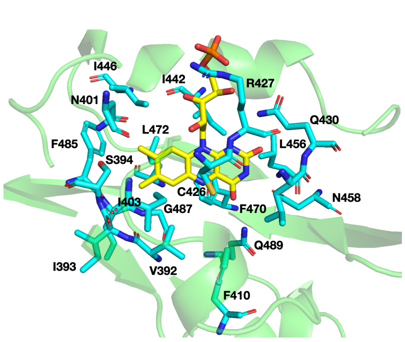

(d) AtLOV2

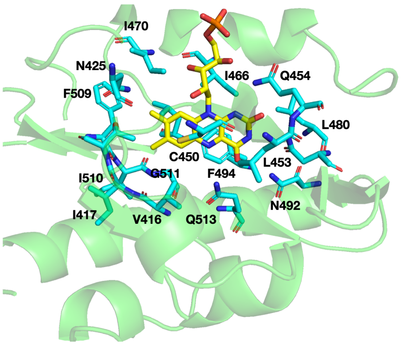

(e) AsLOV2

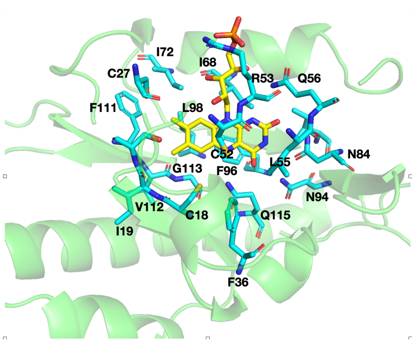

(f) CrLOV2

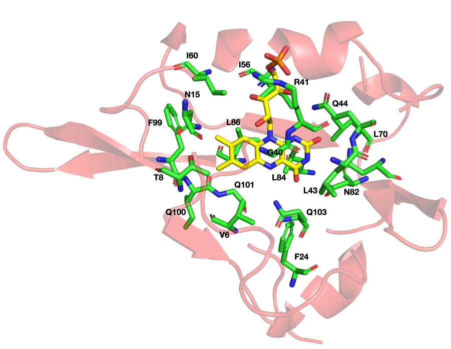

(g) miniSOG

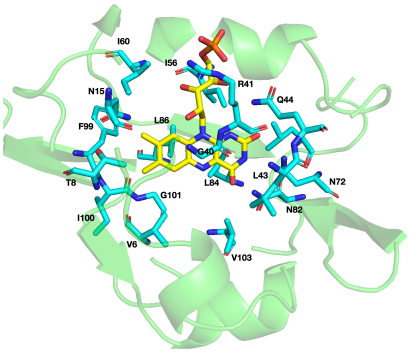

(h) SOPP3

Figure S7: Six LOV domains showing nearby residues within 4Å: (a) AtLOV1, (b) AsLOV1, (c) CrLOV1, (d) AtLOV2, (e) AsLOV2, (f) CrLOV2, (g) miniSOG and (h) SOPP3. LOV1 domains have been represented here with red color and LOV2 domians with green as shown in Figs. 7 and 8 in the main manuscript.

S6 TD-B3LYP/cc-pVTZ PESs of various LOV1 and LOV2 domains: path to $T_{n_{N,\pi^*}}$ state

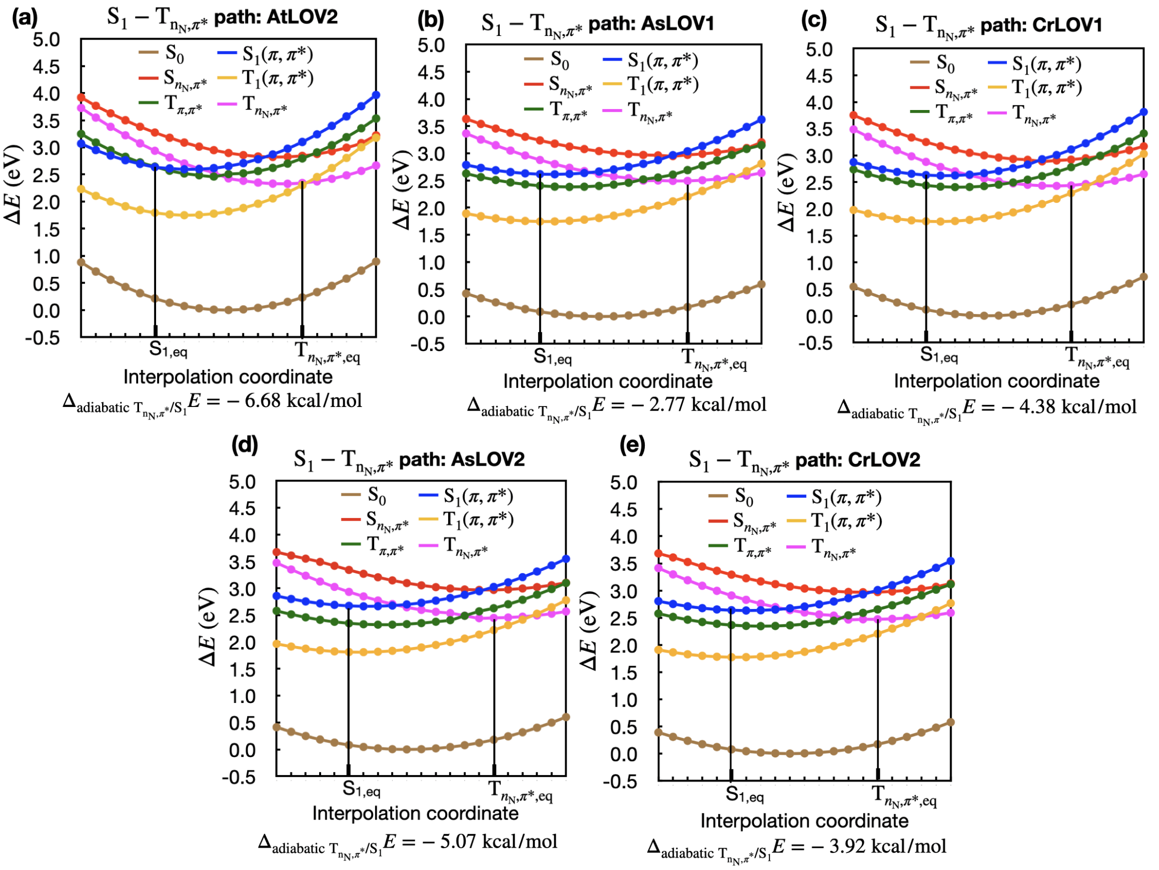

Figure S8: TD-B3LYP/cc-pVTZ PES of LF in various LOV1 and LOV2 domains connecting the $S_{1\pi,\pi^*}$ minimum to T_{n_N,π^*} minimum. In this PES, ground state S_0 (brown), and five low-lying excited states i.e. $S_{1\pi,\pi^*}$ (blue), S_{n_N,π^*} (red), T_1 (yellow), T_{π,π^*} (green) and T_{n_N,π^*} (magenta) have been shown to locate the potential crossings between low-lying excited states. The corresponding $\Delta_{\text{adiabatic } T_{n_N,\pi^*}/S_1}E$ for each LOV domains has been shown below each PES: (a) AtLOV2, (b) AsLOV1, (c) CrLOV1, (d) AsLOV2 and (e) CrLOV2.

S7 Alphafold3 structures of LOV1 and LOV2 domains with pLDDT legend

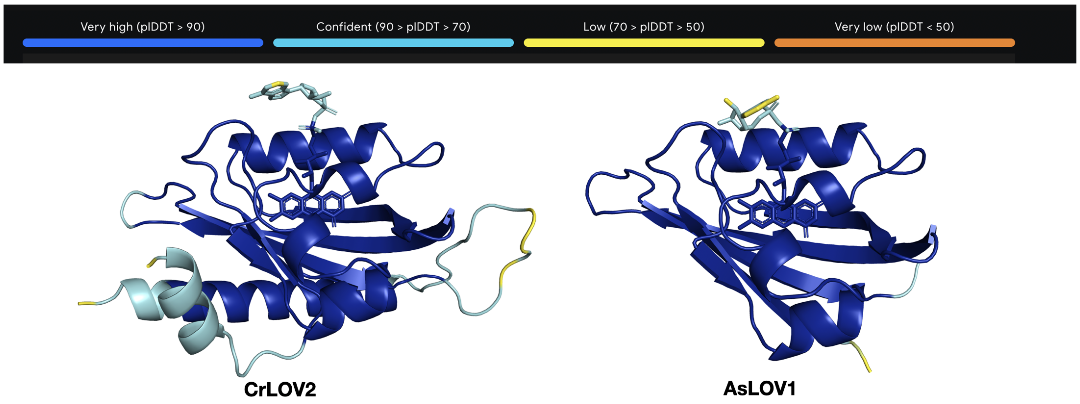

Figure S9: AlphaFold3 predicted structures of CrLOV2 and AsLOV1 with the pLDDT legend.

S8 Full protein sequence

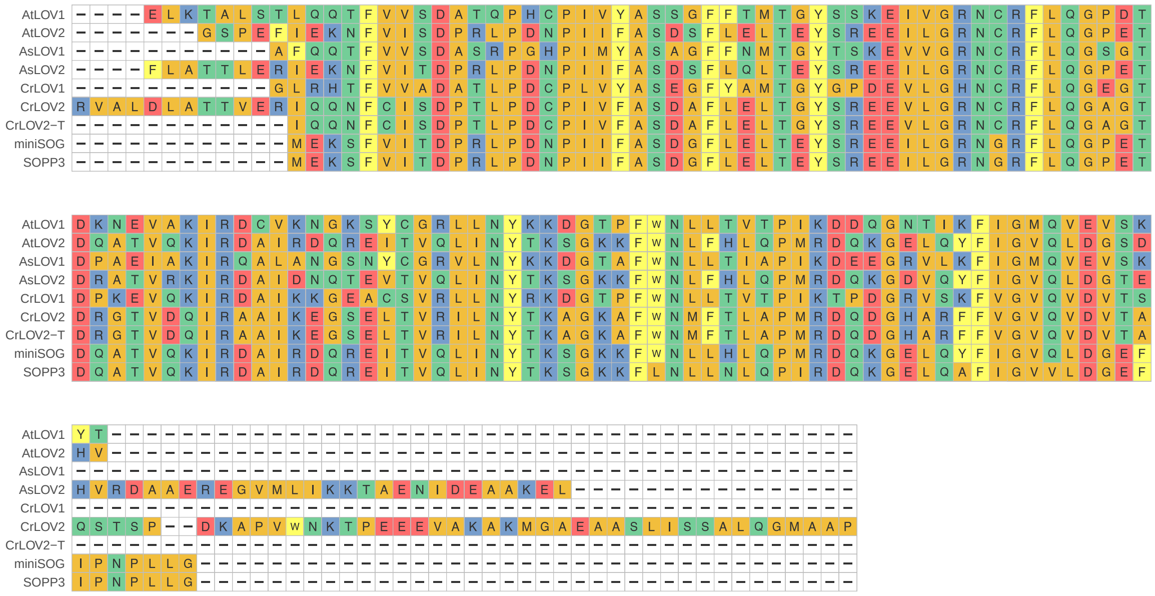

Figure S10: Full sequence of LOV1 and LOV2 domains used in QM/MM calculations. CrLOV2-T i.e., the 108-residue truncated CrLOV domain, is shown to establish reference for Fig. 3 in the main text

S9 Comparison of CrLOV2 models with and without the J'α helix

Protein	8-roots (10,10) MS-CASPT2	15-roots (14,12) MS-CASPT2	
	$S_{1\pi,\pi^*}$ (λ_{max}) in nm	$S_{1\pi,\pi^*}$ (eV)	T_{n_N,π^*} (eV)
CrLOV2 with J'Alpha	430 ± 1	2.73	2.97
CrLOV2-T	430 ± 1	2.72	2.97

Table S1: Comparison of λ_{max} (nm), VEEs (in eV) of $S_{1\pi,\pi^*}$ and T_{n_N,π^*} states of CrLOV2 with J'α and crLOV2-T.

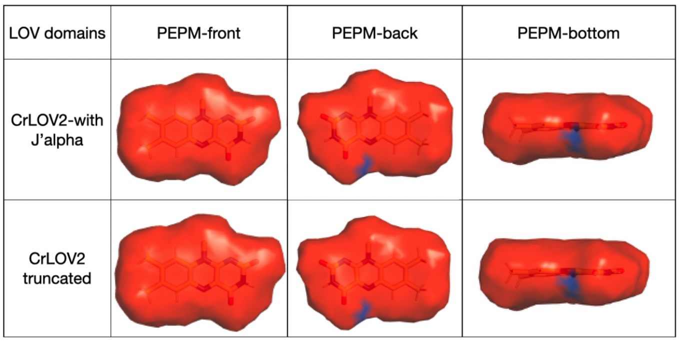

Figure S11: Projection of protein electrostatics onto the Van der Waals surface of LF in CrLOV2 with J'α and in CrLOV2 truncated.
